# A Novel IGLC2 Gene Linked With Prognosis of Triple-Negative Breast Cancer

**DOI:** 10.3389/fonc.2021.759952

**Published:** 2022-01-27

**Authors:** Yu-Tien Chang, Wen-Chiuan Tsai, Wei-Zhi Lin, Chia-Chao Wu, Jyh-Cherng Yu, Vincent S. Tseng, Guo-Shiou Liao, Je-Ming Hu, Huan-Ming Hsu, Yu-Jia Chang, Meng-Chiung Lin, Chi-Ming Chu, Chien-Yi Yang

**Affiliations:** ^1^ School of Public Health, National Defense Medical Center, Taipei, Taiwan; ^2^ Department of Pathology, Tri-Service General Hospital, National Defense Medical Center, Taipei, Taiwan; ^3^ Graduate Institute of Life Sciences, National Defense Medical Center, Taipei, Taiwan; ^4^ Division of Nephrology, Department of Medicine, Tri-Service General Hospital, National Defense Medical Center, Taipei, Taiwan; ^5^ Division of General Surgery, Department of Surgery, Tri-Service General Hospital, National Defense Medical Center, Taipei, Taiwan; ^6^ Department of Computer Science, National Chiao Tung University, Hsinchu, Taiwan; ^7^ Graduate Institute of Medical Sciences, National Defense Medical Center, Taipei, Taiwan; ^8^ Division of Colorectal Surgery, Department of Surgery, Tri-Service General Hospital, National Defense Medical Center, Taipei, Taiwan; ^9^ School of Medicine, National Defense Medical Center, Taipei, Taiwan; ^10^ Department of Surgery, Songshan Branch of Tri-Service General Hospital, National Defense Medical Center, Taipei, Taiwan; ^11^ Graduate Institute of Clinical Medicine, College of Medicine, Taipei Medical University, Taipei, Taiwan; ^12^ Cell Physiology and Molecular Image Research Center, Wan Fang Hospital, Taipei Medical University, Taipei, Taiwan; ^13^ Cancer Research Center and Translational Laboratory, Taipei Medical University Hospital, Taipei Medical University, Taipei, Taiwan; ^14^ Division of Gastroenterology, Department of Medicine, Taichung Armed Forces General Hospital, Taichung, Taiwan; ^15^ Department of Biological Science and Technology, National Chiao Tung University, Hsinchu, Taiwan; ^16^ Division of Biostatistics and Informatics, Department of Epidemiology, School of Public Health, National Defense Medical Center, Taipei, Taiwan; ^17^ Big Data Research Center, Fu-Jen Catholic University, New Taipei City, Taiwan; ^18^ Department of Public Health, China Medical University, Taichung, Taiwan; ^19^ Department of Healthcare Administration and Medical Informatics College of Health Sciences, Kaohsiung Medical University, Kaohsiung, Taiwan

**Keywords:** immunoglobulin, breast cancer, triple-negative breast cancer (TNBC), MDA-MB-231, prognosis, next-generation sequencing, relapse-free survival, distant metastasis-free survival

## Abstract

**Background:**

Immunoglobulin-related genes are associated with the favorable prognosis of triple-negative breast cancer (TNBC) patients. We aimed to analyze the function and prognostic value of immunoglobulin lambda constant 2 (IGLC2) in TNBC patients.

**Methods:**

We knocked down the gene expression of IGLC2 (IGLC2-KD) in MDA-MB-231 cells to evaluate the proliferation, migration, and invasion of tumors *via* 3-(4,5-Dimethythiazol-2-yl)-2,5-diphenyl tetrazolium bromide assay, wound healing, and transwell cell migration assay respectively. Relapse-free survival (RFS) and distant metastasis-free survival (DMFS) analyses were conducted using the KM plotter online tool. The GSE76275 data set was used to analyze the association of IGLC2 and clinical characteristics. A pathway enrichment analysis was conducted using the next-generation sequencing data of wild-type and IGLC2-KD MDA-MB-231 cells.

**Results:**

The low gene expression of IGLC2 was related to unfavorable RFS, DMFS. The high expression of IGLC2 was exhibited in the basal-like immune-activated (BLIA) TNBC molecular subtype, which was immune-activated and showed excellent response to immune therapy. IGLC2 was positively correlated with programmed death-ligand 1 (PD-L1) as shown by Spearman correlation (r = 0.25, p < 0.0001). IGLC2 had a strong prognostic effect on lymph node-negative TNBC (RFS range: 0.31, q value= 8.2e-05; DMFS = 0.16, q value = 8.2e-05) but had no significance on lymph node-positive ones. The shRNA-mediated silencing of IGLC2 increased the proliferation, migration, and invasion of MDA-MB-231 cells. The results of pathway enrichment analysis showed that IGLC2 is related to the PI3K-Akt signaling pathway, MAPK signaling pathway, and extracellular matrix–receptor interaction. We confirmed that MDA-MB-231 tumor cells expressed IGLC2, subverting the traditional finding of generation by immune cells.

**Conclusions:**

IGLC2 linked with the proliferation, migration, and invasion of MDA-MB-231 cells. A high expression of IGLC2 was related to favorable prognosis for TNBC patients. IGLC2 may serve as a biomarker for the identification of TNBC patients who can benefit the most from immune checkpoint blockade treatment.

## Highlights

►Immunoglobulin lambda constant 2 (IGLC2) is a novel prognostic biomarker for TNBC patients.
►The high expression of IGLC2 is related to favorable relapse-free survival, distant metastasis-free survival, tumor size, and TNBC molecular subtypes.
►IGLC2 has a strong prognostic value for lymph node-negative TNBC.
►Silencing of IGLC2 linked with the proliferation, migration, and invasion of MDA-MB-231 cell lines.
►IGLC2 influences TNBC possibly through the pathways of PI3K-Akt signaling, MAPK signaling, and extracellular matrix–receptor interaction.
►IGLC2 is positively associated with programmed death-ligand 1 (PD-L1).

## Introduction

Breast cancer (BC) is the leading cause of cancer and the most common cancer in women worldwide, affecting approximately 12% of females during their lifetimes (1). BC is very heterogenic and classified into distinct molecular subtypes based on hormone receptors, namely, estrogen receptor (ER)/progesterone receptor (PR), and growth factors, including human epidermal growth factor receptor 2 (HER2) and Ki-67 as a proliferation marker (2). Triple-negative BC (TNBC) is a subtype of BC that lacks the expression of ER, PR, and HER-2, is generally aggressive, has high rates of relapse, and results in a decreased overall survival (3, 4). TNBC accounts for 10%–20% of all BCs (5). Given that TNBC lacks the expression of ER/PR/HER2, the application of endocrine therapy or targeted therapy against HER2 is difficult. Chemotherapy has become the main treatment mode, but it generally presents a poor efficacy. Standardized TNBC treatment regimens are still lacking. Therefore, the development of new TNBC treatment strategies has become an urgent clinical need (4). Understanding the molecular profiles of TNBC is critical for the development of new therapeutic options to prevent the progression of metastatic illness and eventually improve the survival of this patient population (6).

TNBC can be categorized into various molecular subtypes, including by the six subtypes by Lehmann (7) or the four subtypes by Burstein (8), based on gene expression profiling of tumor samples (4). Burstein’s four subtypes include luminal androgen receptor (LAR), mesenchymal (MES), basal-like immunosuppressed (BLIS), and basal-like immune-activated (BLIA) subtypes, with BLIA displaying the upregulation of genes controlling B-cell, T-cell, and natural killer cell functions with distinct prognoses (8). The efficacy of existing treatment regimens, therapeutic drugs, and targeted treatment regimens on TNBC subtypes varies (4). Immunotherapies [e.g., programmed cell death protein 1 (PD-1) or Programmed death ligand 1 (PD-L1) inhibitors] may be useful treatments for BLIA tumors, whereas VTCN1-immuno-regulator may be effective treatments for BLIS tumors (9).

Novel biomarkers have been successfully identified by genetic co-expression network (GCN) analysis in BCs (10–24). GCN is an undirected graph, where each node corresponds to a gene, and a pair of nodes is connected with an edge if there is a significant co-expression relationship between them (25). In our previous study (26), we combined the methods of GCN and gene expression profiling to identify six novel immunoglobulin-related gene modules (*IGHA1, IGHD, IGHG1, IGHG3*, immunoglobulin lambda constant 2 (*IGLC2*), and *IGLJ3*) associated with favorable prognosis for TNBC patients. The mRNA expression data sets of 920 BC tumor tissue samples were from the National Center for Biotechnology Information GEO data sets (recurrence n = 354, no recurrence n = 566, and average follow-up time of four data sets = 6–9 years).

*IGLC2* had the most significant effects out of six genes; its hazard ratios (HRs) in relapse-free survival (RFS) and distant metastasis-free survival (DMFS) were 0.64 (p = 0.038) and 0.13 (p = 0.025), respectively. These six immunoglobulin genes were involved in the tumor microenvironment of B lymphocytes, which play important roles in BC prognosis, especially in TNBC (27). Growing evidence indicates that variants of immunoglobulin segments are associated with the prognosis of BC (including TNBC) (28, 29). The stromal immunoglobulin kappa chain (IGKC) serves as an immunologic biomarker of initiation, prognosis (30, 31), and treatment response of BCs and other cancers (32). Among the six discovered immunoglobulin genes, IGLC2 has the most significant effect on prognosis, but only its association with lymphoid neoplasia (33) and amyloidosis has been discussed (34). To the best of our knowledge, no study has evaluated the prognostic effect and function of IGLC2 in TNBC. Therefore, we aimed to analyze the function and prognostic value of IGLC2 in TNBC patients.

## Methods

### Kaplan–Meier (KM) Survival Analysis

We used the KM plotter online cancer survival analysis tool (35) to conduct the Kaplan–Meier plot and survival analysis of IGLC2. The data of breast cancers comprised 55 independent datasets from National Center of Biotechnology Information Gene Expression Omnibus database. The total number of breast cancer arrays was 9,423 and 7,830 unique samples (36). All the available data sets of TNBC were basal-like in the KM plotter online cancer survival analysis tool. The case numbers of RFS and DMFS were 392 and 306, respectively (**Table 1**).

**Table 1:** Description of validation data set from KM plotter for RFS and DMFS analysis of IGLC2.

	TNBC tissues for RFS survival analysis (n = 392)n (%)	TNBC tissues for DMFS survival analysis (n = 306)n (%)
**Molecular subtypes**		
Basal like	392 (1)	306 (1)
**Lymph node status**		
Positive	189 (49)	117 (53)
Negative	197 (51)	102 (47)
**Grade**		
1	15 (5)	2 (1)
2	34 (12)	24 (13)
3	240 (83)	153 (85)

### GSE Data Set

We used GSE76275 (37) to analyze the association of IGLC2 mRNA expression and clinical characteristics using linear regression. We included all the TNBC tissues (n = 198) and variables of age, body mass index, menopause, TNBC molecular subtypes, tumor size, stage, grade, the number of positive nodes, and metastasis. There were 115 TNBC tissues left for analysis after missing data was removed. The pairwise scatter plots and boxplots of IGLC2 with other variables were shown in **Figure S1**. **Table 2** shows the description of GSE76275. The gene expression of whole-genome was converted to the log base 2 of the value before the statistical analysis. After transformation, the distributions of whole-genome expression were close to normal distribution and in a similar range (**Figure S2**).

**Table 2:** Association of IGLC2 mRNA expression and clinical characteristics of TNBC tissues from GSE76275 data set.

	IGLC2 log2 mRNA expression					q values^*^
	Mn	SD	%	n	p value^#^	
**Age (years)**	56	13	100.00%	115	0.17	0.54
**BMI (kg/m^2^)**	28	6	100.00%	115	0.69	0.76
**Race**						
** Asian or Pacific islander**	12.83	3.56	3.5%	4	ref	
** Caucasian**	12.46	1.99	93.0%	107	0.72	0.76
** Missing**	12.51	1.74	3.5%	4		
**Female**	12.47	2.03	100.0%	115		
**Menopause**						
** Pre-menopause**	12.45	1.81	26.1%	30	ref	
** Menopause**	13.27	1.36	3.5%	4	0.43	0.74
** Post-menopause**	12.56	2.04	51.3%	59	0.81	0.81
** Missing**	12.13	2.39	19.1%	22		
**Molecular subtype**						
** Basal-Like Immune-Activated (BLIA)**	13.04	1.78	27.8%	32	ref	
** Basal-Like Immune-Suppressed (BLIS)**	11.93	2.16	31.3%	36	**0.02**	0.20
** Luminal-AR (LAR)**	11.69	1.79	20.0%	23	**0.01**	0.20
** Mesenchymal (MES)**	13.27	1.93	20.9%	24	0.66	0.76
**Tumor size (cm)**						
** ≤2cm**	12.71	1.77	18.3%	21	ref	
** 2-5cm**	12.51	2.10	70.4%	81	0.69	0.76
** >5cm**	12.11	2.07	6.1%	7	0.22	0.54
** Any size with direct extension**	11.54	1.93	5.2%	6	0.50	0.74
**Stage**						
** I**	12.71	1.77	18.3%	21	ref	
** II**	12.51	2.10	70.4%	81	0.69	0.76
** IIIA**	12.11	2.07	6.1%	7	0.50	0.74
** IIIB**	11.54	1.93	5.2%	6	0.22	0.54
**Grade**						
** Well Differentiated**	14.89		0.9%	1	ref	
** Moderately Differentiated**	11.78	1.88	27.0%	31	0.11	0.54
** Poorly Differentiated**	12.75	1.89	57.4%	66	0.26	0.54
**Number of positive nodes**						
** 0**	12.14	2.22	51.3%	59	ref	
** 1-3**	12.84	1.86	32.2%	37	0.10	0.54
** 4-9**	12.88	1.39	9.6%	11	0.27	0.54
** ≥10**	12.63	1.88	7.0%	8	0.52	0.74
**Metastasis**						
** No**	12.51	2.00	98.3%	113	ref	
** Yes**	10.55	3.43	1.7%	2	0.18	0.54

^#^p values of univariable linear regression. ref, reference group; Mn, mean; SD, standard deviation. P < 0.05 was marked in bold.

^*^q values were calculated using Benjamini-Hochberg method. Missing values were not included for statistical analysis.

### Correction for Multiple Comparisons

The Benjamini–Hochberg method was applied to control the False Discovery Rate (FDR) for multiple hypothesis testing (38) and q values were calculated using the R function “stats” of built-in package “stats” (39). The p values and q values were displayed in the tables and figures.

### Next Generation Sequencing (NGS)

One wild type (WT) and two IGLC2 knockdown (IGLC2-KD) cell lines were collected. We used poly-T oligo-attached beads to purify mRNA, which was also fragmented primed for cDNA synthesis, and used a reverse transcriptase and random primer to synthesize the first-strand cDNA and dUTP in place of dTTP to generate a double-stranded (ds) cDNA. A single “A” nucleotide was added to the 3′ end of the ds cDNA. Then, multiple indexing adapters were ligated to the 5′ and 3′ of the ends of the ds cDNA. Polymerase chain reaction (PCR) was used to selectively amplify the DNA fragments that had adapters on both ends. The library was validated on an Agilent 2100 Bio-analyzer and Real-Time PCR System. We conducted NGS following the protocol of Illumina NextSeq sequencing and calculated the gene expression (RSEM, http://deweylab.github.io/RSEM/), differential gene expression (EBSeq, https://www.biostat.wisc.edu/~kendzior/EBSEQ/), pathway enrichment, and Gene Ontology (GO) enrichment.

### Pathway Enrichment Analysis

Pathway enrichment analysis helps researchers gain mechanistic insights into gene lists generated from genome-scale (omics) experiments and identifies biological pathways that are enriched in a gene list more than that would be expected by chance (40). We adopted the method of Gene Set Enrichment Analysis (GSEA) which is a computational method that determines whether an *a priori* defined set of genes shows statistically significant, concordant differences between two biological states (41, 42). Kyoto Encyclopedia of Genes and Genomes (KEGG) and GO pathway enrichment analyses were conducted using differentially expressed genes (DEGs) from the NGS results of IGLC2-KD and WT cells. KEGG is a database resource of high-level functions and utilities of the biological system from large-scale molecular datasets (43). The GO knowledgebase is the world’s largest source of information on the functions of genes, in three aspects of cellular component (CC), biological process (BP), and molecular function (MF) using computational analysis of large-scale molecular biology and genetics experiments (44).

### Cell Culture and Reagents

The human breast carcinoma cell line MDA-MB-231 (RRID: CVCL_0062) was maintained in Roswell Park Memorial Institute-1640 medium (Sigma-Aldrich, St. Louis, MO) supplemented with 10% fetal bovine serum at 37°C in humidified air containing 5% carbon dioxide.

### Knockdown of IGLC2 Expression in MDA-MB-231 Cells

The knockdown of IGLC2 gene in MDA-MB-231 cells was generated using IGLC2-specific shRNA. IGLC2-shRNA-containing lentiviral vectors were purchased from Applied Biological Materials Inc. (#246730910296) and prepared in accordance with standard protocols. The target sequences of IGLC2 were 37 CGCCCTCCTCTGAGGAGCTTCAAGCCAAC, 158 GGAGACCACCACACCCTCCAAACAAAGCA, 197 CGCGGCCAGCAGCTATCTGAGCCTGACGC and 255 AGCTGCCAGGTCACGCATGAAGGGAGCAC. The human breast carcinoma cell line MDA-MB-231 was transinfected with lentiviruses in the selection medium containing 2 µg/ml polybrene. At 48 h after infection, the cells were treated with 10 mg/mL puromycin to select a pool of puromycin-resistant clones. We measured the IGLC2 knock-down efficacy of multiple clones and selected the best one for further experiment (**Figure S3**).

### Immunoblot Analysis

The cells were harvested in lysis buffer (50 mM Tris pH 8.0, 5 mM NaCl, 0.5% NP-40, and 1X protease inhibitor). The protein concentration was determined using the Bradford method (Bio-Rad, Hercules, CA). Samples with an equivalent amount of protein were loaded onto a sodium dodecyl sulfate-polyacrylamide gel and electrophoresed. The separated proteins were transferred to a nitrocellulose membrane. Then, the membrane was probed anti-IGLC2 antibody (IGLC2 monoclonal antibody (5E12B9), ThermoFisher Scientific, MA5-31776), followed by a secondary antibody in phosphate-buffered saline (PBS)/Tween 20 with 5% Carnation nonfat milk. Proteins were detected using an enhanced chemiluminescence reagent (ECL Plus, GE).

### MTT Assay

3-(4,5-Dimethythiazol-2-yl)-2,5-diphenyl tetrazolium bromide (MTT) assay was used to evaluate cell proliferation. Cells were seeded at a density of 1x10^4^ cells/well in 24-well plates. Subsequently, the MTT reagent (Sigma) was added to each well, and the plates were incubated for 1 h at 37°C. The remaining crystals were dissolved in a mixture medium consisting of 100 µl dimethyl sulfoxide and 100 µl 95% alcohol (1:1). The crystals were shaken on a shaker for 10–15 min until dissolution. The absorbance was evaluated at OD_540–570_ using an enzyme-linked immunosorbent assay reader. The assays were performed in triplicate. The significance was calculated using Student’s t-test.

### Wound-Healing Assay

Wound-healing assay was used to evaluate cell migration. Cells were seeded in six-well plates at 1x10^5^ cells per well in a growth medium. Confluent monolayers were starved overnight in assay medium, and a single scratch was created. The cells were washed with PBS to remove cell debris, supplemented with assay medium, and monitored. Images were captured under a microscope at 0 and 27 h post-wounding.

### Transwell Cell Migration Assay

The migratory ability was evaluated in a BD Falcon cell culture insert (BD Biosciences). Aliquots of 1 × 10^5^ cells suspended in 500 µl serum-free media were seeded into the upper part of each chamber, and the lower compartments were filled with media containing 10% FCS. After incubation for 24–72 h, nonmigrating cells were physically removed from the upper surface of the membrane. The MDA-MB-231 cells were stained using 0.2% crystal violet. The MDA-MB-231 cells were counted in at least 10 randomly fields per insert at 100x magnification.

## Results

### IGLC2 Had a Beneficial Effect on TNBC Patients

**Figure 1:**
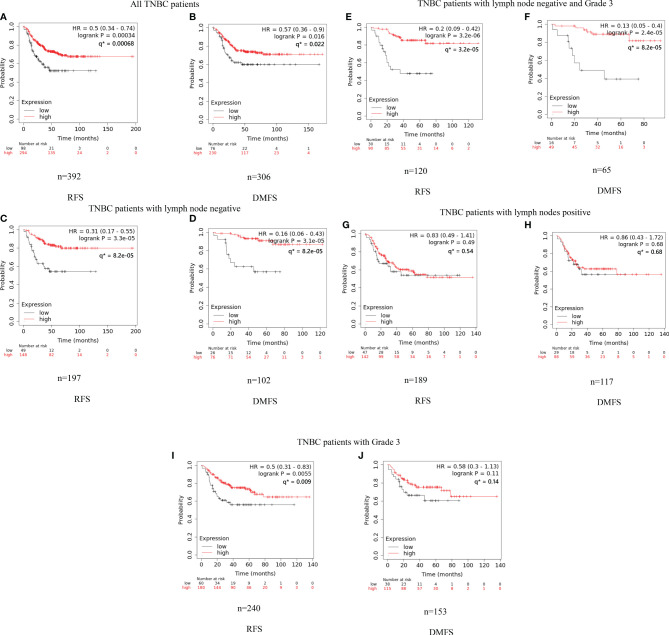
Kaplan–Meier analysis of RFS and DMFS of IGLC2 mRNA expression for TNBC subgroups. We used the KM plotter online cancer survival analysis tool (http://kmplot.com/analysis/) to evaluate the RFS **(A, C, E, G, I)** and DMFS **(B, D, F, H)** in TNBC subgroups grouped by grade and lymph node status. The panels **(A, B)** are all from TNBC patients; **(C, D)** are TNBC patients developing negative lymph nodes; **(E, F)** are TNBC patients developing negative lymph nodes and Grade 3; **(G, H)** are TNBC patients developing positive lymph nodes; **(I, J)** are TNBC patients developing Grade 3. The lower quartile was set to be the cut-off point of IGLC2 gene expression. Grade 1 and 2 subgroups were not analyzed given the limited sample size (n < 35) for meaningful analysis. Y axis denotes the probability of RFS or DMFS. * q values were calculated using Benjamini-Hochberg method.

**Figure 2:**
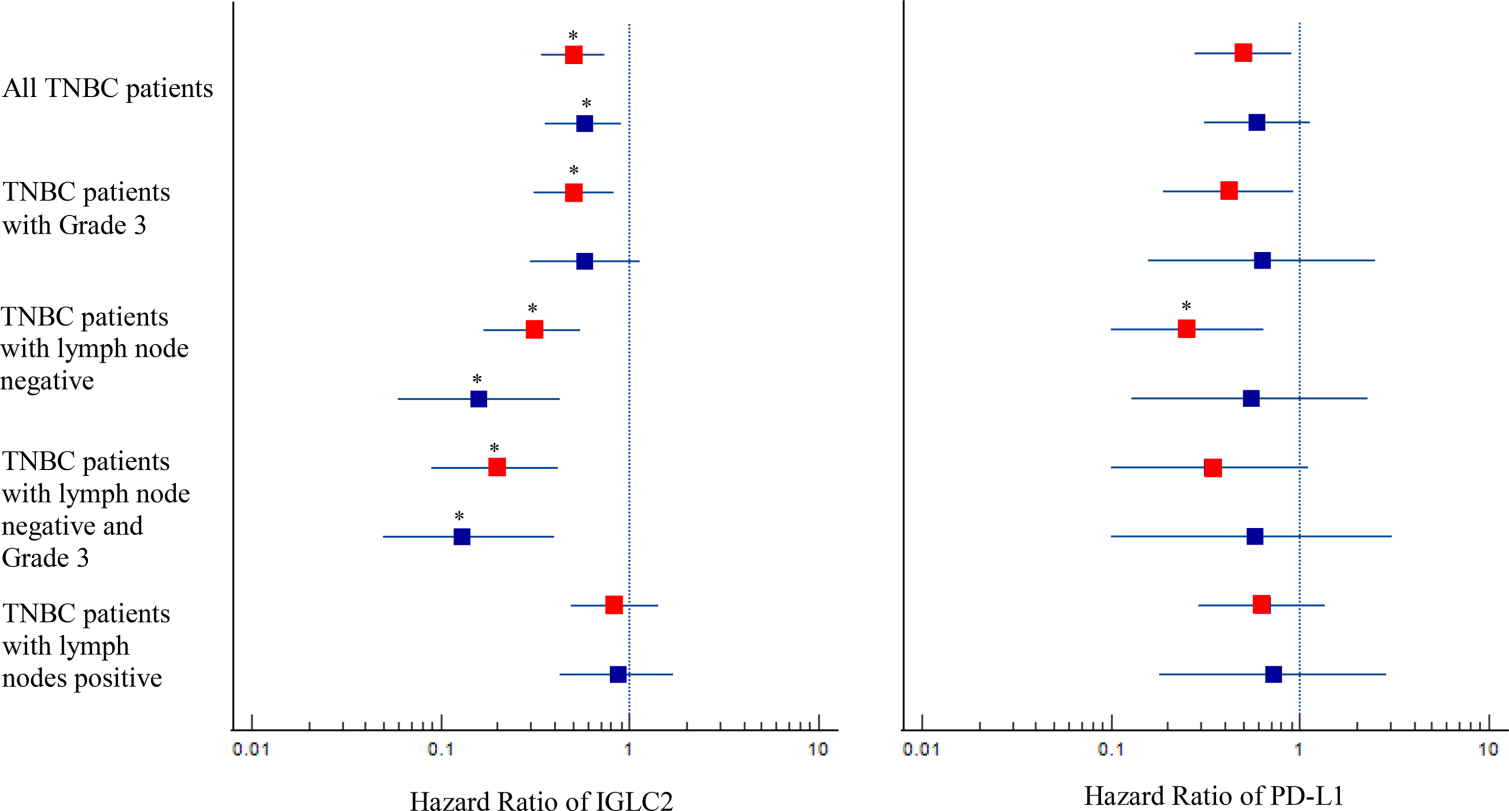
Forest plot with hazard ratio (HR) for IGLC2 and PD-L1 of Kaplan–Meier (KM) survival analysis of RFS and DMFS. The HRs were obtained from the KM plotter online tool. The analysis data were grouped as a whole, by lymph node status, and by Grade 3. The boxplot was the 95% CI of HR. A 95% CI of HR not equal to 1 denotes statistical significance before multiple comparison adjustment. The HR results of RFS were marked in red, and those of DMFS were marked in blue. The correction of multiple comparisons was using Benjamini-Hochberg method and q values less than 0.05 were marked with an asterisk (*).

We conducted the survival analysis of IGLC2 mRNA expression of TNBC tissue samples with 392 RFS and 306 DMFS (**Table 1**) *via* the KM plotter online tool (35). The TNBC molecular subtypes of this data were all basal like. The proportion of TNBC patients with negative or positive lymph node was similar in the analysis data of RFS and DMFS (**Table 1**). Over 80% of the TNBC tissues were Grade 3. In the sensitivity analysis of prognosis, the TNBC tissues were analyzed as a whole and divided into three subgroups by Grade 3 and lymph node status (negative and positive). The cut-off point of IGLC2 gene expression was set to be the lower quartile rather than the median (**Figure S4**) for better prediction results. A high mRNA expression of IGLC2 was associated with improved RFS and DMFS in TNBC patients (**Figures 1A, B**). IGLC2 is a great prognostic gene, especially for TNBC patients developing lymph node-negative (**Figures 1C, D**) and lymph node-negative with Grade 3 (**Figures 1E, F**), compared with all TNBC patients (**Figures 1A, B**), lymph node-positive TNBC patients (**Figures 1G, H**) and Grade 3 TNBC patients (**Figures 1I, J**). Meanwhile, IGLC2 was not a significant prognostic predictor of RFS and DMFS for lymph node-positive TNBC (**Figures 1G, H**). For Grade 3 TNBC (**Figures 1G, H** and **Figure 2**), IGLC2 was not a significant prognostic predictor of DMFS. IGLC2 presented a better prognostic value for DMFS than RFS in TNBC patients developing lymph node-negative, although no statistical significance was found (**Figure 2**). No survival analysis was conducted for patients developing Grades 1 and 2 TNBC due to the limited sample number (less than 35) (**Table 1**) for meaningful survival analysis.

In addition, we used TCGA-BRCA gene expression data of TNBC (n=131) to validate IGLC2 in our previous study (26). Because there was no corresponding gene IGLC2 in TCGA-BRCA microarrays, we validated all related immunoglobulin genes: IGLL3, IGLL1, IGSF9B, IGDCC3, IGDCC4, IGBP1, IGSF5, IGSF11, IGSF22, IGSF21, IGHMBP2, IGSF10, IGSF8, IGSF9, IGSF6, IGSF1, IGSF3, IGFN1, and IGJ. The results showed that IGDCC3 and IGSF3 were significantly associated with RFS.

### Association of *IGLC2* and Clinical Characteristics

**Figure 3:**
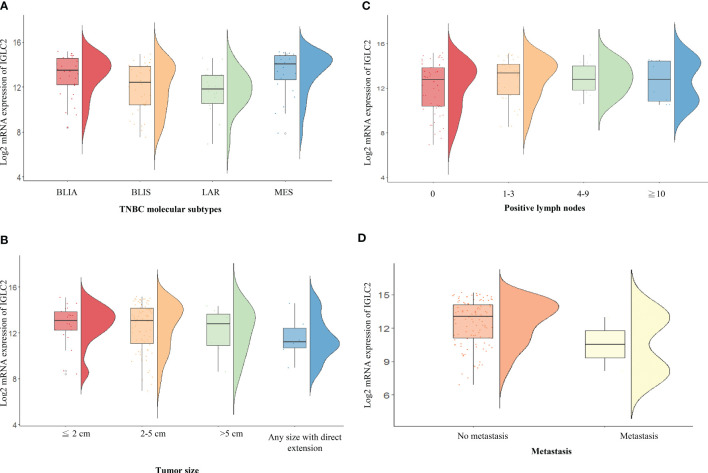
RainCloud plots of IGLC2 mRNA expression grouped by **(A)** TNBC molecular subtypes [BLIA, BLIS, LAR, and MES], **(B)** tumor size (≤2, 2–5, and >5 cm), **(C)** the number of positive lymph nodes (0, 1–3, 4–9, and ≥10), and **(D)** metastasis (no and yes) using the GSE76275 data set.

We analyzed the association of IGLC2 and clinical characteristics using the mRNA expression data set of 115 TNBC tissues from GSE76275 (37). The IGLC2 mRNA expression was associated with the TNBC molecular subtypes but not significant after the adjustment of multiple comparisons (**Table 2**). The mRNA expression of IGLC2 was higher in BLIA and MES molecular subtypes of TNBC compared with BLIS and LAR (**Figure 3A**).

A low IGLC2 mRNA expression was exhibited in large tumor-size tissues; especially in tumors of any size with direct extension (**Figure 3B**). The expression of IGLC2 was lower in Stage IIIB patients compared with that in Stage I patients (**Table 2**). IGLC2 expression decreased from TNBC patients with positive lymph nodes 1-3 to ≥ 10 (**Table 2** and **Figure 3C**). The IGLC2 expression was lower in TNBC patients with metastasis than those without metastasis (**Figure 3D**).

### Association of *IGLC2*, PD-1, and Programmed Death-Ligand 1 (PD-L1)

Immune checkpoint blockade is a promising treatment for TNBC. However, the selection of patients who will benefit the most remains a challenge. PD-L1 expression is widely used as a predictive biomarker due to its association with desirable response rates to PD1/PD-L1 blockade for TNBC patients (45). Therefore, we analyzed the association of IGLC2, PD-1, and PD-L1 to unveil the potential of IGLC2 as a biomarker for identifying TNBC patients who can benefit from immune checkpoint blockade. IGLC2 and PD-L1 were positively correlated, with r = 0.25 (p value < 0.0001) in Spearman correlation (**Figure S5**).

**Figure 4:**
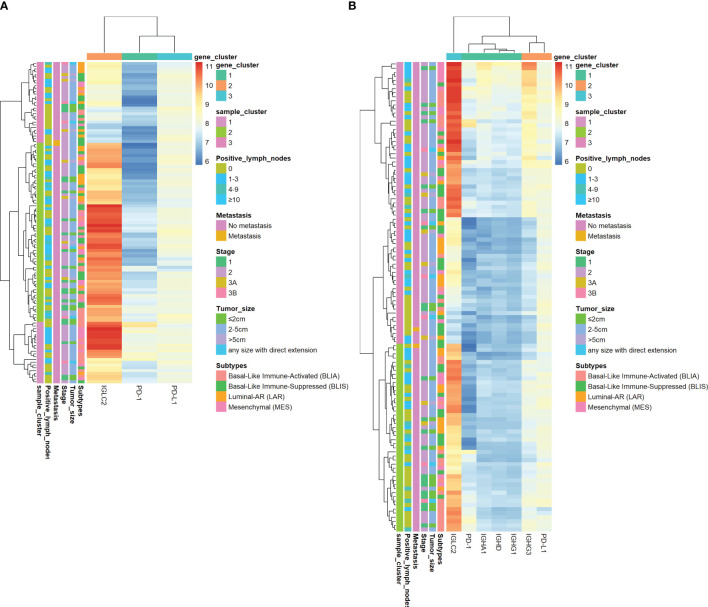
Heatmap of log2 mRNA expression of IGLC2, PD-1, and PD-L1 of TNBC tissues using GSE 76275. **(A)**The heatmap of IGLC2, PD-1, and PD-L1, **(B)** The heatmap of IGLC2, other highly co-expressed immunoglobulin genes found in our previous study (26), PD-1, and PD-L1.

**Figure 4** shows the heatmap of the mRNA expressions of IGLC2, other highly co-expressed immunoglobulin genes of IGLC2 found in our previous study (26), PD-1, and PD-L1. The IGLC2 expression was more variable in TNBC samples than those of co-expressed immunoglobulin genes and PD-1, PD-L1. In addition, IGLC2 was more relevant to the RFS and DMFS of TNBC patients than PD-L1 (**Figure 2**, **Figure S6** and **Table S1**). The lower expression of IGLC2 was associated with unfavorable tumor size and metastasis although there was no statistical significance (**Figure 3**). Since IGLC2 was related to PD-L1 and more specific to the clinical phenotypes of TNBC patients compared with PD-L1, IGLC2 may be a potential biomarker for the identification of TNBC patients who can benefit the most from immune checkpoint blockade treatment as well as a prognostic biomarker.

### Role of *IGLC2* in Tumor Cell Proliferation

**Figure 5:**
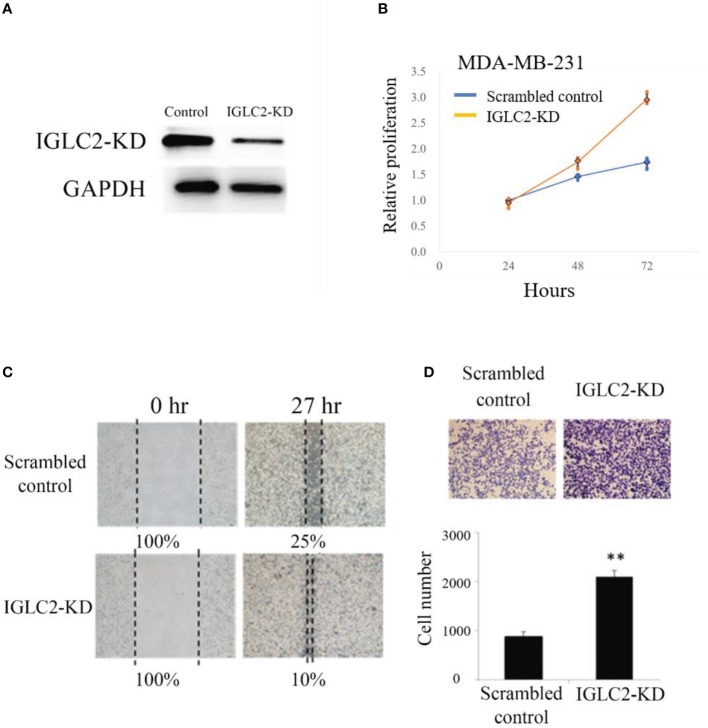
Knockdown of expression of IGLC2 increased proliferation, migration, and healing of MDA-MB-231 cells. **(A)** Immunoblot analysis of IGLC2-KD in MDA-MB-231 cells. Glyceraldehyde-3-phosphate dehydrogenase was used as an internal control. **(B)** The proliferation ability of IGLC2-KD MDA-MB-231 cells increased compared with that of the scrambled control in the MTT assay. **(C)** In the wound-healing migratory assay, IGLC2-KD MDA-MB-231 cells showed a faster healing ability than the scrambled control cells. **(D)** The migratory ability of IGLC2-KD MDA-MB-231 cells increased compared with that of the scrambled control in the transwell cell migration assay. The MDA-MB-231 cells were dyed by crystal violet staining. Statistical analysis was performed using Mann–Whitney U test. **P < 0.01 was considered significantly different.

We knocked down the gene expression of IGLC2 (IGLC2-KD) in MDA-MB-231 cell lines using short hairpin RNA (shRNA) and stably transfected cells. The protein expression of IGLC2 decreased and was confirmed in the immunoblot analysis (**Figure 5A**). MTT assay was used to ascertain the role of *IGLC2* in tumor proliferation. The silencing of *IGLC2* significantly increased the proliferation of MDA-MB-231 cell lines at 48 and 72 h (p < 0.01) (**Figure 5B**).

### Silencing *IGLC2* Influenced the Migratory and Invasive Abilities of MDA-MB-231 Cell Lines

Cell mobility is a key indicator of malignant tumor progression. Metastasis is also an important issue in clinical therapeutics. The wound-healing ability of *IGLC2*-KD cells increased compared with that of scrambled control cells in the MDA-MB-231 cell line (**Figure 5C**). We analyzed the role of *IGLC2* in tumor migration using the transwell cell migration assay. As shown in **Figure 5D**, the migratory behavior of cells significantly increased (p < 0.01) after the knockdown of IGLC2 in MDA-MB-231 cell lines. These results revealed that *IGLC2* mediates the migratory ability of MDA-MB-231 cell lines. Concerning the potential association of cell death and IGLC2, we analyzed the correlation of IGLC2 and well-known cell cycle genes (CDK2, CDK4, CDK6, CCNA1, CCNB1 and CCND1) (46) using GSE76275 data set (37) (**Figure S7**). IGLC2 was significantly associated with CDK6 and CCNA1 with the correlation coefficients r -0.19 and 0.17 (p<0.05) using Spearman correlation (**Figure S8**). The mechanism of cell death induced by IGLC2 warrants further study.

### Pathway Enrichment Analysis

**Figure 6:**
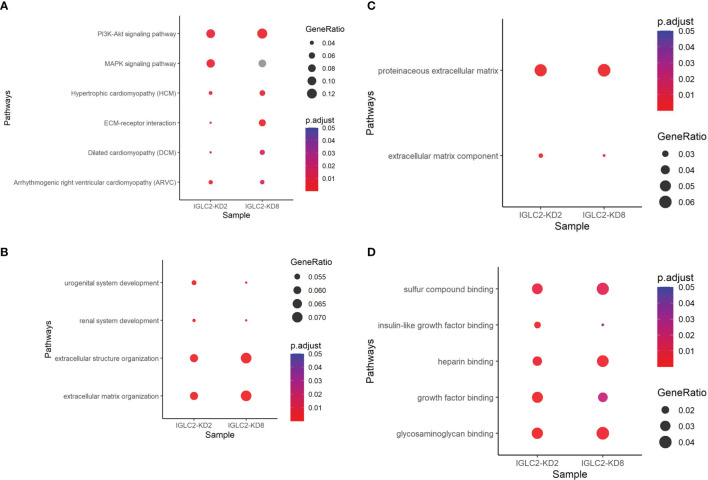
Dot plots of pathway enrichment analysis using annotations of **(A)** KEGG, **(B)** GO-BP, **(C)** GO-CC, and **(D)** GO-MF. P.adjust means adjusted p value of pathway enrichment analysis. GeneRatio denotes the percentage of target genes existing in the enriched pathway.

To understand the possible pathways related to IGLC2, we conducted NGS of three MDA-MB-231 cell lines, which included one wild-type (WT) and two IGLC2-KD MDA-MB-231 cell lines (IGLC2-KD#2 and IGLC2-KD#8) with various knockdown degrees. We observed 341 and 191 differentially expressed genes (DEGs) in IGLC2-KD#2 and IGLC2-KD#8 cells compared with WT cells, respectively. We computed the pathway enrichment analysis of the abovementioned DEGs using KEGG and GO annotations. Six enriched KEGG pathways (**Figure 6A** and **Table S2**) were recorded: phosphatidylinositol-3 kinase (PI3K)-Akt signaling pathway, mitogen-activated protein kinase (MAPK) signaling pathway, arrhythmogenic right ventricular cardiomyopathy (ARVC), hypertrophic cardiomyopathy (HCM), dilated cardiomyopathy (DCM), and extracellular matrix (ECM)–receptor interaction. The GO-enriched pathways included urogenital system development, ECM organization, and renal system development in the BP database (**Figure 6B** and **Table S3**); proteinaceous ECM and ECM components in the CC database (**Figure 6C** and **Table S4**); glycosaminoglycan binding, growth factor binding, heparin binding, insulin-like growth factor binding, and sulfur compound binding in the MF database (**Figure 6D** and **Table S5**). Overall, IGLC2 may influence the metastasis of TNBC through the pathways related to ECM organization and cell binding, including glycosaminoglycan, growth factors, heparin, insulin-like growth factor, and sulfur compound binding.

## Discussion

To our knowledge, this research is the first study to confirm the function and beneficial prognostic effect of IGLC2 on the progression of TNBC. The knockdown of IGLC2 expression increased the proliferation, migration, and invasion of MDA-MB-231 cells. A high IGLC2 gene expression increased the RFS and DMFS in TNBC patients. The prognostic value of IGLC2 was stronger in predicting DMFS than RFS. IGLC2 had a strong prognostic value for lymph node-negative TNBC patients but had no significant effect on lymph node-positive and Grade 3 TNBC ones. IGLC2 was expressed highly in BLIA molecular subtype which was significant with unadjusted p value but became insignificant after correction for multiple comparisons. The results of pathway enrichment analysis indicated that IGLC2 may influence the proliferation and metastasis of TNBC through the PI3K-Akt, MAPK, and ECM–receptor interaction pathways.

The mRNA expressions of IGLC2 and PD-L1 were positively correlated. IGLC2 was more specific to TNBC prognosis, molecular subtypes, and clinical phenotypes than PD-1/PD-L1. Thus, IGLC2 has potential as a prognostic biomarker for the identification of TNBC patients who can benefit the most from immune therapy of PD-1/PD-L1 inhibitor agents. In addition, we confirmed that IGLC2 was expressed in MDA-MB-231 cells rather than simply generated by immune cells, as mentioned in previous studies (26).

IGLC2 is a protein-coding gene. Its protein constitutes the constant region of the immunoglobulin heavy chain. IGLC2 shows a potentially high mutation burden in a pan-cancer context (24, 47). Evidence indicates that the overexpression of immunoglobulin gene signatures (48) leads to a better prognosis of overall survival and disease-free survival in the ER−/HER2− subgroup and TNBCs (49). Other studies showed that the overexpression of a seven-gene module (*C1QA, XCL2, SPP1, TNFRSF17, LY9, IGLC2*, and *HLA-F*) has a better prognosis in ER-negative BCs. The downregulation of this module confers a high risk of distant metastasis (HR: 2.02, p = 0.009) that is independent of lymph node status and lymphocytic infiltration (50). Bianchini’s team (51) discovered that B-cell/plasma metagene dominated by immunoglobulin *(IGL, IGKC, IGHC3 IGHA1*, and *IGHG3*) has an independently prognostic value in ER-negative BCs, indicating that a high B-cell/plasma metagene score is correlated to favorable DMFS. We observed that IGLC2 and IGKC were highly correlated with each other with a Pearson correlation r close to 1 (**Figure S9**). IGKC is an independent prognostic biomarker for TNBC patients and a marker of the humoral immune system (31). Tumor-infiltrating plasmablasts and plasma cells were identified as sources of IGKC expression (52). Therefore, we speculated that IGLC2 is a biomarker of the humoral immune system and associated with tumor-infiltrating lymphocytes (TILs). High expressions of IGLC2 reflect the activated humoral immune system.

Immunoglobulin genes are generally regarded to be produced by plasma or B cells rather than tumor cells (53, 54). In this study, IGLC2 was also expressed in tumor cells and played important roles in the prognosis of TNBC. This finding may explain the controversial effects of immunoglobulins on TNBC; Yang (55) stated that high immunoglobulin expression in BCs is correlated with the malignancy and American Joint Committee on Cancer stages of cancers (55), which are contradictory to our findings. This situation implies that various types and origins of immunoglobulins may exert different roles in cancers and thus warrants further study to understand the mechanisms of tumor cells. Growing evidence has indicated that immunoglobulins are not only produced by mature B lymphocytes or plasma cells but also by various normal cell types at immune privileged sites and neoplasms, including non-hematopoietic human cancer cells (55–57) and BC (55). Babbage’s team (53) found rearranged V_H_ transcripts in the most commonly used BC cell lines. They guessed that BC tumor cells *in vivo* acquired extraneous genes from neighboring cells and kept them in their genome, or malignant epithelial cells may have initiated the required cascade of complex molecular events to rearrange the VH genes (53).

Rakha’s team observed that tumor size, lymph node stage, and androgen receptor are clinical prognostic markers in TNBC (58). In the lymph node-positive subgroup, the size and androgen receptor retained their prognostic significance. However, in the lymph node-negative tumor subgroup, basal phenotype is the sole prognostic marker identified (58). Thus, the prognostic factors of TNBC varied with lymph node status. Molecular subtypes played major roles in the prognosis of lymph node-negative TNBC patients. We observed that IGLC2 had a strong beneficial prognostic effect on lymph node-negative TNBCs but had no significant influence on lymph node-positive ones. In addition, the IGLC2 expression was associated with TNBC molecular subtypes. Our finding echoed Rakha’s results.

We also observed the high expression levels of PD-1 in lymph node-positive TNBC (**Figure S10**). PD-1 plays a vital role in inhibiting immune responses. The PD-1/PD-L1 axis inhibits T-cell activation, proliferation, and survival and cytotoxic secretion within cancer cells (59). We speculated that the immune response was suppressed in lymph node-positive TNCB. Thus, IGLC2 no longer reflected the effective prognostic effect of immune response against tumors. Therefore, no significant prognostic value was observed with IGLC2 in lymph node-positive TNBC.

The tumor microenvironment is a complex system formed by distinct and interacting cell populations, and its composition is related to cancer prognosis and response to clinical treatment (60). Immune modules were predictive of the pathological complete response to neoadjuvant chemotherapy in ER-/HER2-BCs (48, 61–63). B cells are present and activated in approximately one quarter of BCs and represent up to 40% of the TIL population in several BCs (49). The success of monoclonal antibody-based immunotherapy indicates the potential for harnessing the humoral immune response in BC treatment (64–67). Immunotherapy has become a promising treatment for TNBC. However, the immune checkpoint inhibitor monotherapy targeting PD-1/PD-L1 shows a mild response for TNBC patients (68). Nevertheless, promising results were found in the combination treatment of immune checkpoint inhibitors and chemotherapy in clinical trials (69). PD-L1 is the only biomarker applied in clinical practice for the selection of patients who are likely to respond to PD-1/PD-L1 immune checkpoint inhibitors. The elevated PD-L1 expression of TNBC tumors predicted an improved response to PD-1/PD-L1 immune checkpoint inhibitor treatment (45). However, the definition of “PD-L1-positive” population in clinical practice remains challenging (69). In addition, assessing whether a cancer is “immune activated” or “immune inactivated” is difficult (70). Therefore, finding new molecular biomarkers for the prediction immunotherapy response for TNBC is an urgent issue.

TNBC is the subtype of BCs most related to TIL infiltration and PD-1/PD-L1 expression (70). Among the TNBC subtypes, BLIA is enriched in immune-response genes, immune activated, and can benefit the most from immune checkpoint inhibitor treatment (69). We observed that the IGLC2 expression was significantly associated with PD-L1 expression and expressed in higher levels in BLIA subtypes.

He’s team identified three TNBC subtypes, namely, Immunity High (Immunity_H), Immunity Medium (Immunity_M), and Immunity Low (Immunity_L) subtypes. Immunity_H is characterized by greater immune cell infiltration and anti-tumor immune activities and better survival prognosis compared with the other subtypes. A high immunity is positivity associated with PD-L1 levels (71). We speculated that IGLC2 is a biomarker of immune cell infiltration and anti-tumor immune activities. Therefore, we observed the positive correlation between IGLC2 and PD-L1. In addition, IGLC2 and PD-1/PD-L1 belong to the immunoglobulin superfamily, and IGLC2 is more specific to TNBC. Compared with PD-1/PD-L1, IGLC2 was more correlated with the prognosis of TNBC patients and more variable with TNCB clinical phenotypes. Thus, IGLC2 may be a potential biomarker for the identification of TNBC patients who are immune activated and will benefit from the current immune therapy.

The results of GO pathway enrichment analysis of IGLC2 indicated that IGLC2 may influence the migration of TNBC *via* the pathways of receptor binding and ECM organization. Insulin-like growth factor binding was one of the notable pathways identified. Insulin-like growth factor binding protein-3 (IGFBP-3) drives an oncogenic pathway in human TNBC cell lines (7) involving the activation of tyrosine kinase receptor epidermal growth factor receptor (EGFR) and lipid kinase sphingosine kinase (SphK) (72) and is associated with poor prognosis (73). IGFBP-3 promotes the growth of TNBC cells by increasing the EGFR signaling, which is mediated by SphK1, and the combined inhibition of EGFR and SphK1 has potential as an anticancer therapy in TNBC in which EGFR and IGFBP-3 expression is high (74).

In the results of KEGG pathway enrichment analysis, IGLC2 may influence the progression of TNBC *via* the PI3K-Akt signaling pathway, MAPK signaling pathway, ECM–receptor interaction, HCM, DCM, and ARVC. The findings of MAPK signaling and PI3K-Akt signaling were the same with He’s study, that is, these pathways are hyperactivated in TNBC subtype with high immunity (71). The PI3K/AKT/mammalian target of rapamycin pathway is the most frequently altered pathway in BC (75) and TNBC (76, 77). This pathway has been studied to identify promising new targets for the treatment of TNBC (76, 78–80). Ras-MAPK pathway activation promotes immune evasion and is related to the resistance to conventional chemotherapy in TNBC (81). Several studies have analyzed the targeted inhibitors of the Ras/MAPK pathway to identify potential treatment targets in TNBC (81–85). Increasing evidence emphasizes the crucial role of the ECM in BC progression, invasion, and metastasis (86). The ECM–receptor interaction pathway is associated with a poor prognosis, high metastatic risk (87, 88), and high incidence of chemotherapy resistance of BCs (89). Cardiomyopathy is a common adverse effect of chemotherapeutic agents (i.e., trastuzumab and doxorubicin) for BC (90–92), which may partially explain why IGLC2 is related to HCM, DCM, and ARVC. IGLC2 may serve as a potential biomarker to monitor or reduce cardiomyopathy in BC chemotherapy.

There were some limitations in the study. We only used one TNBC cell line MDA-MB-231 to validate IGLC2. The biological processes related with cell death involved by IGLC2 should be analyzed. Thus, we used multiple independent mRNA data sets from TNBC tissues to support our findings. Our team is still working on the functional analysis of IGLC2. The abovementioned limitations will be included in our future work.

## Conclusions

The suppression of IGLC2 gene expression increases the proliferation and migration of MDA-MB-231 cell lines. The function pathways may be involved in PI3K-Akt, MAPK, and ECM–receptor interaction. The high expression of IGLC2 mRNA was related to favorable RFS and DMFS of TNBC patients. IGLC2 exhibited a strong beneficial prognostic effect on lymph node-negative TNBC patients but had no prognostic value for lymph node-positive TNBC patients. The combination evaluation of IGLC2 and clinical lymph node status can provide a precise prognosis prediction of TNBC patients. IGLC2 is positively correlated with PD-L1 and specific to TNBC. IGLC2 may be a potential biomarker for identifying TNBC patients who can benefit the most from immune checkpoint blockade treatment.

## Data Availability Statement

Publicly available datasets were analyzed in this study. This data can be found here: The validation data from the mRNA microarray and survival information are available in the online tool KM plotter (http://kmplot.com/analysis/). GSE76275 was from NCBI GEO database available at https://www.ncbi.nlm.nih.gov/geo.

## Author Contributions

Data curation, Y-TC; Formal analysis, Y-TC, Y-JC and W-ZL; Funding acquisition, C-YY, M-CL and J-MH; Investigation, Y-TC and Y-JC; Methodology, Y-TC and W-ZL; Resources, G-SL, C-YY, J-MH, C-MC, M-CL, H-MH and V-ST; Supervision, C-MC; Validation, Y-TC; Writing – original draft, Y-TC; Writing – review & editing, Y-TC, W-CT, G-SL, Y-JC, M-CL, J-MH, C-CW, G-SL, J-CY, C-MC and H-MH.

## Funding

This work was supported by the Songshan Branch of Tri-Service General Hospital, National Defense Medical Center [grant numbers 807SB109602] and Taichung Armed Forces General Hospital [grant numbers TCAFGH-E-110040].

## Conflict of Interest

The authors declare that the research was conducted in the absence of any commercial or financial relationships that could be construed as a potential conflict of interest.

## Publisher’s Note

All claims expressed in this article are solely those of the authors and do not necessarily represent those of their affiliated organizations, or those of the publisher, the editors and the reviewers. Any product that may be evaluated in this article, or claim that may be made by its manufacturer, is not guaranteed or endorsed by the publisher.

## Abbreviations

TNBC, triple-negative breast cancer; BC, breast cancer; GCNA, genetic co-expression network analysis; RFS, relapse-free survival; DMFS, distant metastasis-free survival; DEGs, differentially expressed genes.
