# Supplementary material for: A Novel IGLC2 Gene Linked With Prognosis of Triple-Negative Breast Cancer
Source: Front Oncol. 2022 Jan 27;11:759952. doi: 10.3389/fonc.2021.759952 (PMC8829566; doi:10.3389/fonc.2021.759952)
Supplement: Supplementary file 1 [file DataSheet_1.docx]

**A Novel IGLC2 Gene Linked with Prognosis of Triple-Negative Breast Cancer**

Yu-Tien Chang^1^, greengarden720925@gmail.com

Wen-Chiuan Tsai^2^, [ab95057@hotmail.com](mailto:ab95057@hotmail.com)

Wei-Zhi Lin^3^, [wz.lin@gapps.ndmctsgh.edu.tw](mailto:wz.lin@gapps.ndmctsgh.edu.tw)

Chia-Chao Wu^4^, [wucc@mail.ndmctsgh.edu.tw](mailto:wucc@mail.ndmctsgh.edu.tw)

Jyh-Cherng Yu^5^, doc20106@mail.ndmctsgh.edu.tw

Vincent S Tseng^6^, vtseng@cs.nctu.edu.tw

Guo-Shiou Liao^5*^, liaogs20371@gmail.com

Je-Ming Hu^7,8,9*^, jeminghu@gmail.com

Huan-Ming Hsu^5,10*^, hmh0823@gmail.com

Yu-Jia Chang^11,12,13*^, [r5424012@tmu.edu.tw](mailto:r5424012@tmu.edu.tw)

Meng-Chiung Lin^15,16*^, [s88.s99@msa.hinet.net](mailto:s88.s99@msa.hinet.net)

Chi-Ming Chu^17,18,19,20 *^, [cm.chu.tw@gmail.com](mailto:cm.chu.tw@gmail.com)

Chien-Yi Yang^10*^,[wayneyoung680324@gmail.com](mailto:wayneyoung680324@gmail.com)

*Equal contribution

**Authors’ affiliations:**

^1^School of Public Health, National Defense Medical Center, Taipei, Taiwan.

^2^Department of Pathology, Tri-Service General Hospital, National Defense Medical Center, Taipei, Taiwan

^3^ Graduate Institute of Life Sciences, National Defense Medical Center, Taipei City, Taiwan

^4^Division of Nephrology, Department of Medicine, Tri-Service General Hospital, National Defense Medical Center, Taipei, Taiwan

^5^Division of General Surgery, Department of Surgery, Tri-Service General Hospital, National Defense Medical Center, Taipei, Taiwan

^6^Department of Computer Science, National Chiao Tung University, Hsinchu, Taiwan.

^7^Graduate Institute of Medical Sciences, National Defense Medical Center, Taipei, Taiwan

^8^Division of Colorectal Surgery, Department of Surgery, Tri-Service General Hospital,  National Defense Medical Center, Taipei City, Taiwan

^9^ School of Medicine, National Defense Medical Center, Taipei City, Taiwan

^10^Department of Surgery, Songshan Branch of Tri-Service General Hospital, National Defense Medical Center, Taipei, Taiwan

^11^ Graduate Institute of Clinical Medicine, College of Medicine, Taipei Medical University, Taipei, Taiwan

^12^Cell Physiology and Molecular Image Research Center, Wan Fang Hospital, Taipei Medical University, Taipei, Taiwan

^13^Cancer Research Center and Translational Laboratory, Taipei Medical University Hospital, Taipei Medical University, Taipei, Taiwan

^14^ Division of Gastroenterology, Department of Medicine, Taichung Armed Forces General Hospital, Taichung, Taiwan.

^15^Department of Biological Science and Technology, National Chiao Tung University, Hsinchu, Taiwan

^16^Division of Biostatistics and Informatics, Department of Epidemiology, School of Public Health, National Defense Medical Center, Taipei, Taiwan

^17^Big Data Research Center, Fu-Jen Catholic University, New Taipei City, Taiwan

^18^Department of Public Health, China Medical University, Taichung City, Taiwan

^19^Department of Healthcare Administration and Medical Informatics College of Health Sciences, Kaohsiung Medical University, Kaohsiung, Taiwan

**Corresponding authors:**

Ph.D. Dr. Huan-Ming Hsu

Tel.: +886-2-87923100#18438

Address: No.161, Sec. 6, Minquan E. Rd., Neihu Dist., Taipei City 11490, Taiwan (R.O.C.)

Email: hmh0823@gmail.com

## Table S 1 Association of PD-L1 and PD-1 mRNA expression and clinical characteristics of TNBC tissues from the GSE76275 data set.

|  | **PD-L1 log2 mRNA expression** | | | |  |  |  | **PD-1 log2 mRNA expression** | | | |  |  |
| --- | --- | --- | --- | --- | --- | --- | --- | --- | --- | --- | --- | --- | --- |
|  | Mn | SD | % | n | p value^#^ | q values* |  | Mn | SD | % | n | p value^#^ | q values* |
| **Age (years)** | 56 | 13 | 100.00% | 115 | 0.67 | 0.94 |  | 56 | 13 | 100.00% | 115 | 0.49 | 0.70 |
| **BMI (kg/m^2^)** | 28 | 6 | 100.00% | 115 | 0.67 | 0.94 |  | 28 | 6 | 100.00% | 115 | 0.89 | 0.98 |
| **Race** |  |  |  |  |  |  |  |  |  |  |  |  |  |
| Asian or Pacific islander | 8.05 | 0.18 | 3.5% | 4 | Ref |  |  | 6.47 | 0.17 | 3.5% | 4 | ref |  |
| Caucasian | 8.06 | 0.22 | 93.0% | 107 | 0.91 | 0.94 |  | 6.45 | 0.34 | 93.0% | 107 | 0.93 | 0.98 |
| Missing | 7.90 | 0.36 | 3.5% | 4 |  |  |  | 6.21 | 0.26 | 3.5% | 4 |  |  |
| **Female** | 8.06 | 0.22 | 100.0% | 115 |  |  |  | 6.45 | 0.33 | 100.0% | 115 |  |  |
| **Menopause** |  |  |  |  |  |  |  |  |  |  |  |  |  |
| Pre-menopause | 8.03 | 0.21 | 26.1% | 30 | ref |  |  | 6.44 | 0.31 | 26.1% | 30 | ref |  |
| Menopause | 8.14 | 0.14 | 3.5% | 4 | 0.30 | 0.94 |  | 6.33 | 0.30 | 3.5% | 4 | 0.55 | 0.73 |
| Post-menopause | 8.08 | 0.20 | 51.3% | 59 | 0.22 | 0.94 |  | 6.39 | 0.35 | 51.3% | 59 | 0.49 | 0.70 |
| Missing | 8.03 | 0.30 | 19.1% | 22 |  |  |  | 6.63 | 0.26 | 19.1% | 22 |  |  |
| **Molecular subtype** |  |  |  |  |  |  |  |  |  |  |  |  |  |
| Basal-Like Immune-Activated (BLIA) | 8.06 | 0.30 | 27.8% | 32 | ref |  |  | 6.70 | 0.31 | 27.8% | 32 | ref |  |
| Basal-Like Immune-Suppressed (BLIS) | 8.07 | 0.17 | 31.3% | 36 | 0.87 | 0.94 |  | 6.30 | 0.30 | 31.3% | 36 | **1.07E-07** | **2.1E-06** |
| Luminal-AR (LAR) | 7.99 | 0.22 | 20.0% | 23 | 0.23 | 0.94 |  | 6.26 | 0.28 | 20.0% | 23 | **2.07E-07** | **2.1E-06** |
| Mesenchymal (MES) | 8.11 | 0.14 | 20.9% | 24 | 0.38 | 0.94 |  | 6.50 | 0.23 | 20.9% | 24 | 0.01 | 0.07 |
| **Tumor size (cm)** |  |  |  |  |  |  |  |  |  |  |  |  |  |
| ≤2cm | 8.06 | 0.26 | 18.3% | 21 | ref |  |  | 6.44 | 0.35 | 51.3% | 59 | ref |  |
| 2-5cm | 8.05 | 0.21 | 70.4% | 81 | 0.87 | 0.94 |  | 6.44 | 0.31 | 32.2% | 37 | 0.40 | 0.70 |
| >5cm | 8.12 | 0.14 | 6.1% | 7 | 0.48 | 0.94 |  | 6.49 | 0.39 | 9.6% | 11 | 0.45 | 0.70 |
| any size with direct extension | 8.14 | 0.29 | 5.2% | 6 | 0.44 | 0.94 |  | 6.48 | 0.29 | 7.0% | 8 | 0.46 | 0.70 |
| **Stage** |  |  |  |  |  |  |  |  |  |  |  |  |  |
| I | 8.06 | 0.26 | 18.3% | 21 | ref |  |  | 6.51 | 0.38 | 18.3% | 21 | ref |  |
| II | 8.05 | 0.21 | 70.4% | 81 | 0.87 | 0.94 |  | 6.44 | 0.32 | 70.4% | 81 | 0.40 | 0.70 |
| IIIA | 8.12 | 0.14 | 6.1% | 7 | 0.48 | 0.94 |  | 6.40 | 0.09 | 6.1% | 7 | 0.45 | 0.70 |
| IIIB | 8.14 | 0.29 | 5.2% | 6 | 0.44 | 0.94 |  | 6.39 | 0.52 | 5.2% | 6 | 0.46 | 0.70 |
| **Grade** |  |  |  |  |  |  |  |  |  |  |  |  |  |
| Well Differentiated | 7.98 |  | 0.9% | 1 | ref |  |  | 6.79 |  | .9% | 1 | ref |  |
| Moderately Differentiated | 7.99 | 0.25 | 27.0% | 31 | 0.94 | 0.94 |  | 6.36 | 0.38 | 27.0% | 31 | 0.23 | 0.70 |
| Poorly Differentiated | 8.07 | 0.20 | 57.4% | 66 | 0.67 | 0.94 |  | 6.47 | 0.33 | 57.4% | 66 | 0.37 | 0.70 |
| Missing | 8.12 | 0.21 | 14.8% | 17 |  |  |  | 6.47 | 0.27 | 14.8% | 17 |  |  |
| **Number of positive nodes** |  |  |  |  |  |  |  |  |  |  |  |  |  |
| 0 | 8.07 | 0.21 | 51.3% | 59 | ref |  |  | 6.44 | 0.35 | 51.3% | 59 | ref |  |
| 1-3 | 8.06 | 0.26 | 32.2% | 37 | 0.84 | 0.94 |  | 6.44 | 0.31 | 32.2% | 37 | 0.98 | 0.98 |
| 4-9 | 8.03 | 0.20 | 9.6% | 11 | 0.60 | 0.94 |  | 6.49 | 0.39 | 9.6% | 11 | 0.65 | 0.81 |
| ≥10 | 8.04 | 0.12 | 7.0% | 8 | 0.76 | 0.94 |  | 6.48 | 0.29 | 7.0% | 8 | 0.72 | 0.85 |
| **Metastasis** |  |  |  |  |  |  |  |  |  |  |  |  |  |
| No | 8.06 | 0.22 | 98.3% | 113 | ref |  |  | 6.45 | 0.33 | 98.3% | 113 | ref |  |
| Yes | 8.08 | 0.16 | 1.7% | 2 | 0.90 | 0.94 |  | 5.92 | 0.34 | 1.7% | 2 | 0.02 | 0.10 |

^#^ p values of univariable linear regression. ref: reference group, Mn: mean, SD: standard deviation. Missing values were not included for statistical analysis. * q values were calculated using Benjamini-Hochberg method.

## Table S 2 Kyoto Encyclopedia of Genes and Genomes (KEGG) pathway enrichment of IGLC2-KD2 using 341 DEGs and IGLC2-KD8 using 191 DEGs in total.

| Sample | KEGG.ID | Description | Count | GeneRatio | p value | p.adjust | Gene |
| --- | --- | --- | --- | --- | --- | --- | --- |
| IGLC2-KD2 | hsa04010 | MAPK signaling pathway | 31 | 31/341 | 1.06E-05 | 0.001474 | NTRK2//DUSP4//MYC//RASGRP3//ANGPT1//GADD45B//CACNA1D//NGF//CACNG7//GADD45A//TGFB2//CSF1//EGF//PDGFRA//CACNG8//RASGRP1//IL1R1//IL1A//PLA2G4A//DUSP2//FGF1//DUSP1//AREG//PDGFRB//MAPK11//RELB//GNG12//IGF1R//CACNA2D3//PLA2G4B//FGFR4 |
| IGLC2-KD8 | hsa04010 | MAPK signaling pathway | 15 | 15/191 | 0.0084 | 0.095652 | NGF//IL1B//PDGFRA//CSF1R//DDIT3//CACNG8//CACNA1A//RASGRP1//GADD45B//DUSP4//HSPA6//CACNG7//FGF1//PRKCG//GADD45A |
| IGLC2-KD2 | hsa04151 | PI3K-Akt signaling pathway | 34 | 34/341 | 2.70E-05 | 0.002504 | ITGB4//NTRK2//SPP1//CREB5//MYC//COL1A2//ANGPT1//NGF//RELN//GHR//ITGA6//ITGA1//CSF1//EGF//PDGFRA//COL4A1//PPP2R2B//ITGA11//CREB3L1//ITGB3//IL6//LAMA4//FN1//PPP2R2C//FGF1//AREG//IL7R//PDGFRB//JAK2//ITGA8//GNG12//IGF1R//LPAR2//FGFR4 |
| IGLC2-KD8 | hsa04151 | PI3K-Akt signaling pathway | 24 | 24/191 | 1.00E-05 | 0.00085 | COL1A1//COL1A2//RELN//NGF//ITGB4//CREB3L1//PDGFRA//CSF1R//IL6//DDIT4//GNG7//ITGA2//LAMA4//FGF1//ITGB8//SPP1//MYB//ITGA10//TNXB//COMP//COL4A1//IL7R//LAMC3//IL7 |
| IGLC2-KD2 | hsa04512 | ECM-receptor interaction | 12 | 12/341 | 0.000312 | 0.014446 | ITGB4//SPP1//COL1A2//RELN//ITGA6//ITGA1//COL4A1//ITGA11//ITGB3//LAMA4//FN1//ITGA8 |
| IGLC2-KD8 | hsa04512 | ECM-receptor interaction | 13 | 13/191 | 1.00E-07 | 3.08E-05 | COL1A1//COL1A2//RELN//ITGB4//ITGA2//LAMA4//ITGB8//SPP1//ITGA10//TNXB//COMP//COL4A1//LAMC3 |
| IGLC2-KD2 | hsa05410 | Hypertrophic cardiomyopathy (HCM) | 13 | 13/341 | 8.67E-05 | 0.004818 | ITGB4//CACNA1D//CACNG7//ITGA6//TGFB2//ITGA1//CACNG8//TTN//ITGA11//ITGB3//IL6//ITGA8//CACNA2D3 |
| IGLC2-KD8 | hsa05410 | Hypertrophic cardiomyopathy (HCM) | 10 | 10/191 | 5.00E-05 | 0.002767 | TTN//ITGB4//CACNG8//IL6//ITGA2//CACNG7//ITGB8//DMD//ITGA10//ACE |
| IGLC2-KD2 | hsa05412 | Arrhythmogenic right ventricular cardiomyopathy (ARVC) | 14 | 14/341 | 3.51E-06 | 0.000975 | ITGB4//DSP//CDH2//CACNA1D//CACNG7//ITGA6//ITGA1//CACNG8//GJA1//ITGA11//ITGB3//ITGA8//CACNA2D3//DSG2 |
| IGLC2-KD8 | hsa05412 | Arrhythmogenic right ventricular cardiomyopathy (ARVC) | 8 | 8/191 | 0.0005 | 0.012189 | ITGB4//CACNG8//DSP//ITGA2//CACNG7//ITGB8//DMD//ITGA10 |
| IGLC2-KD2 | hsa05414 | Dilated cardiomyopathy (DCM) | 12 | 12/341 | 0.000743 | 0.029485 | ITGB4//CACNA1D//CACNG7//ITGA6//TGFB2//ITGA1//CACNG8//TTN//ITGA11//ITGB3//ITGA8//CACNA2D3 |
| IGLC2-KD8 | hsa05414 | Dilated cardiomyopathy (DCM) | 9 | 9/191 | 0.0005 | 0.012189 | TTN//ITGB4//CACNG8//ITGA2//CACNG7//ITGB8//DMD//ITGA10//ADCY5 |

P.adjust means adjusted p value of pathway enrichment analysis. GeneRatio denotes the percentage of target genes existing in the enriched pathway.

## Table S 3 Gene Ontology (GO) Biological Process (BP) pathway enrichment of IGLC2-KD2 using 341 DEGs and IGLC2-KD8 using 191 DEGs in total.

| Sample | GO.Term | Description | Count | GeneRatio | pvalue | p.adjust | Gene |
| --- | --- | --- | --- | --- | --- | --- | --- |
| IGLC2-KD2 | GO:0001655 | urogenital system development | 39 | 39/732 | 2.98E-09 | 2.24E-06 | ACTA2//ITGB4//LGR5//SULF1//TP63//MYOCD//MYC//PODXL//ANGPT1//CRLF1//JAG1//STRA6//ITGA6//TGFB2//PDGFRA//AR//SFRP1//NOTCH3//COL4A1//PCSK9//SULF2//HES1//ROBO2//ZNF354A//FGF1//RARB//SMAD7//PDGFRB//ADAMTS1//DCN//ITGA8//KIRREL3//PTPRO//EPHA4//KIF26B//BASP1//SPRY1//HPGD//FMN1 |
| IGLC2-KD8 | GO:0001655 | urogenital system development | 21 | 21/407 | 2.52E-05 | 0.003518 | ACTA2//SULF1//TP73//ITGB4//PDGFRA//ROBO2//PAX2//SIM1//LHX1//CRLF1//BMP4//SPRY1//FGF1//PTCH1//DLL1//COL4A1//ACE//DCHS2//OSR1//FOXC1//CD24 |
| IGLC2-KD2 | GO:0030198 | extracellular matrix organization | 45 | 45/732 | 1.30E-12 | 3.28E-09 | ADAMTS2//BGN//COL5A3//ITGB4//POSTN//SULF1//CRISPLD2//SFRP2//CTGF//SPP1//CYR61//COL1A2//FAP//ITGA6//TGFB2//HAS3//ITGA1//FERMT1//COL4A1//FLRT2//SULF2//ITGA11//CREB3L1//LCP1//ITGB3//LAMA4//FN1//CCDC80//TNFRSF11B//ERCC2//JAM3//FBLN1//COL27A1//COL3A1//DCN//PHLDB2//COL18A1//ITGA8//LOXL3//ITGB2//CARMIL2//LOXL1//COL14A1//FGFR4//COL12A1 |
| IGLC2-KD8 | GO:0030198 | extracellular matrix organization | 29 | 29/407 | 6.01E-10 | 1.26E-06 | COL1A1//COL1A2//SULF1//TNFRSF11B//LCP1//ITGB4//CREB3L1//ICAM1//COL5A2//POSTN//BGN//SFRP2//ABI3BP//ITGA2//LAMA4//ACAN//ITGB8//SPP1//ITGA10//TNXB//CTGF//COMP//COL4A1//LAMC3//TLL1//FOXC1//ELF3//ICAM5//COL19A1 |
| IGLC2-KD2 | GO:0043062 | extracellular structure organization | 45 | 45/732 | 1.45E-12 | 3.28E-09 | ADAMTS2//BGN//COL5A3//ITGB4//POSTN//SULF1//CRISPLD2//SFRP2//CTGF//SPP1//CYR61//COL1A2//FAP//ITGA6//TGFB2//HAS3//ITGA1//FERMT1//COL4A1//FLRT2//SULF2//ITGA11//CREB3L1//LCP1//ITGB3//LAMA4//FN1//CCDC80//TNFRSF11B//ERCC2//JAM3//FBLN1//COL27A1//COL3A1//DCN//PHLDB2//COL18A1//ITGA8//LOXL3//ITGB2//CARMIL2//LOXL1//COL14A1//FGFR4//COL12A1 |
| IGLC2-KD8 | GO:0043062 | extracellular structure organization | 29 | 29/407 | 6.47E-10 | 1.26E-06 | COL1A1//COL1A2//SULF1//TNFRSF11B//LCP1//ITGB4//CREB3L1//ICAM1//COL5A2//POSTN//BGN//SFRP2//ABI3BP//ITGA2//LAMA4//ACAN//ITGB8//SPP1//ITGA10//TNXB//CTGF//COMP//COL4A1//LAMC3//TLL1//FOXC1//ELF3//ICAM5//COL19A1 |
| IGLC2-KD2 | GO:0072001 | renal system development | 38 | 38/732 | 3.44E-10 | 3.88E-07 | ACTA2//ITGB4//LGR5//SULF1//TP63//MYOCD//MYC//PODXL//ANGPT1//CRLF1//JAG1//STRA6//ITGA6//TGFB2//PDGFRA//SFRP1//NOTCH3//COL4A1//PCSK9//SULF2//HES1//ROBO2//ZNF354A//FGF1//RARB//SMAD7//PDGFRB//ADAMTS1//DCN//ITGA8//KIRREL3//PTPRO//EPHA4//KIF26B//BASP1//SPRY1//HPGD//FMN1 |
| IGLC2-KD8 | GO:0072001 | renal system development | 21 | 21/407 | 4.19E-06 | 0.002325 | ACTA2//SULF1//TP73//ITGB4//PDGFRA//ROBO2//PAX2//SIM1//LHX1//CRLF1//BMP4//SPRY1//FGF1//PTCH1//DLL1//COL4A1//ACE//DCHS2//OSR1//FOXC1//CD24 |

P.adjust means adjusted p value of pathway enrichment analysis. GeneRatio denotes the percentage of target genes existing in the enriched pathway.

## Table S 4 Gene Ontology (GO) Cellular Component (CC) pathway enrichment of IGLC2-KD2 using 341 DEGs and IGLC2-KD8 using 191 DEGs in total.

| Sample | GO.Term | Description | Count | GeneRatio | pvalue | p.adjust | Gene |
| --- | --- | --- | --- | --- | --- | --- | --- |
| IGLC2-KD2 | GO:0005578 | proteinaceous extracellular matrix | 45 | 45/768 | 7.19E-11 | 3.21E-08 | ADAMTS2//ALPL//ANOS1//BGN//CHL1//COL5A3//POSTN//CRISPLD2//ADAMTS15//EFEMP1//CTGF//SBSPON//SERPINA1//COL22A1//TIMP3//SMOC1//COL1A2//MGP//EFEMP2//RELN//ITGA6//ADAMTS10//SFRP1//COL4A1//FLRT2//PHOSPHO1//EMID1//LAMA4//FN1//CCDC80//FGF1//TNFRSF11B//FBLN7//FBLN1//FLRT1//COL27A1//COL3A1//ADAMTS1//DCN//COL18A1//LOXL1//COL14A1//DGCR6//MATN2//COL12A1 |
| IGLC2-KD8 | GO:0005578 | proteinaceous extracellular matrix | 27 | 27/438 | 2.06E-07 | 7.41E-05 | COL1A1//COL1A2//RELN//MGP//TNFRSF11B//EMILIN1//COL5A2//POSTN//FREM2//MUC4//BGN//BMP4//ABI3BP//SBSPON//LAMA4//ACAN//FGF1//WNT5B//USH2A//TNXB//CTGF//COMP//COL4A1//CTHRC1//TIMP3//LAMC3//COL19A1 |
| IGLC2-KD2 | GO:0044420 | extracellular matrix component | 20 | 20/768 | 1.28E-07 | 2.34E-05 | COL5A3//TIMP3//SMOC1//COL1A2//EFEMP2//ITGA6//ADAMTS10//COL4A1//LAMA4//FN1//CCDC80//FBLN1//COL27A1//COL3A1//ADAMTS1//COL18A1//LOXL1//COL14A1//MATN2//COL12A1 |
| IGLC2-KD8 | GO:0044420 | extracellular matrix component | 11 | 11/438 | 0.000147 | 0.005879 | COL1A1//COL1A2//EMILIN1//COL5A2//FREM2//LAMA4//USH2A//TNXB//COL4A1//TIMP3//LAMC3 |

P.adjust means adjusted p value of pathway enrichment analysis. GeneRatio denotes the percentage of target genes existing in the enriched pathway.

## Table S 5 Gene Ontology (GO) Molecular Function (MF) pathway enrichment of IGLC2-KD2 using 341 DEGs and IGLC2-KD8 using 191 DEGs in total.

| Sample | GO.Term | Description | Count | GeneRatio | pvalue | p.adjust | Gene |
| --- | --- | --- | --- | --- | --- | --- | --- |
| IGLC2-KD2 | GO:0005539 | glycosaminoglycan binding | 25 | 25/723 | 1.53E-06 | 0.000192 | ANOS1//BGN//CEMIP//COL5A3//POSTN//CRISPLD2//ADAMTS15//CTGF//CYR61//SFRP1//LAYN//FN1//CCDC80//FGF1//PCSK6//APLP2//FBLN7//RTN4RL1//LIPG//ADAMTS1//DCN//DPYSL3//GPNMB//FGFR4//SUSD5 |
| IGLC2-KD8 | GO:0005539 | glycosaminoglycan binding | 17 | 17/405 | 6.8E-06 | 0.00133 | PCSK6//RTN4RL1//SERPIND1//ADA2//POSTN//PCOLCE//BGN//BMP4//ABI3BP//ACAN//FGF1//PTCH1//TNXB//CTGF//COMP//RSPO2//LTF |
| IGLC2-KD2 | GO:0019838 | growth factor binding | 24 | 24/723 | 7.90E-10 | 2.97E-07 | IGFBP3//IGFBP4//IGFBP5//ITGB4//NTRK2//IGFBP1//CTGF//HTRA3//NTRK3//CYR61//COL1A2//ITGA6//PDGFRA//CRIM1//COL4A1//IL1R1//ITGB3//PCSK6//DUSP1//COL3A1//PDGFRB//IGF1R//TNFRSF8//FGFR4 |
| IGLC2-KD8 | GO:0019838 | growth factor binding | 11 | 11/405 | 0.000248 | 0.020812 | COL1A1//COL1A2//PCSK6//IGFBP1//ITGB4//PDGFRA//WISP1//SCN5A//IGFBPL1//CTGF//COL4A1 |
| IGLC2-KD2 | GO:0008201 | heparin binding | 19 | 19/723 | 2.52E-05 | 0.001985 | ANOS1//COL5A3//POSTN//CRISPLD2//ADAMTS15//CTGF//CYR61//SFRP1//FN1//CCDC80//FGF1//PCSK6//APLP2//FBLN7//RTN4RL1//LIPG//ADAMTS1//GPNMB//FGFR4 |
| IGLC2-KD8 | GO:0008201 | heparin binding | 15 | 15/405 | 3.63E-06 | 0.001064 | PCSK6//RTN4RL1//SERPIND1//ADA2//POSTN//PCOLCE//BMP4//ABI3BP//FGF1//PTCH1//TNXB//CTGF//COMP//RSPO2//LTF |
| IGLC2-KD2 | GO:0005520 | insulin-like growth factor binding | 12 | 12/723 | 5.26E-10 | 2.97E-07 | IGFBP3//IGFBP4//IGFBP5//ITGB4//IGFBP1//CTGF//HTRA3//CYR61//ITGA6//CRIM1//ITGB3//IGF1R |
| IGLC2-KD8 | GO:0005520 | insulin-like growth factor binding | 5 | 5/405 | 0.00047 | 0.033836 | IGFBP1//ITGB4//WISP1//IGFBPL1//CTGF |
| IGLC2-KD2 | GO:1901681 | sulfur compound binding | 23 | 23/723 | 7.22E-05 | 0.004523 | ANOS1//COL5A3//POSTN//CRISPLD2//ADAMTS15//CTGF//CYR61//FST//SFRP1//SCP2//GSTM1//FN1//CCDC80//FGF1//PCSK6//APLP2//FBLN7//RTN4RL1//LIPG//ADAMTS1//DPYSL3//GPNMB//FGFR4 |
| IGLC2-KD8 | GO:1901681 | sulfur compound binding | 16 | 16/405 | 8.42E-05 | 0.00901 | PCSK6//RTN4RL1//SERPIND1//ADA2//POSTN//PCOLCE//BMP4//ABI3BP//FGF1//PTCH1//TNXB//FST//CTGF//COMP//RSPO2//LTF |

P.adjust means adjusted p value of pathway enrichment analysis. GeneRatio denotes the percentage of target genes existing in the enriched pathway.


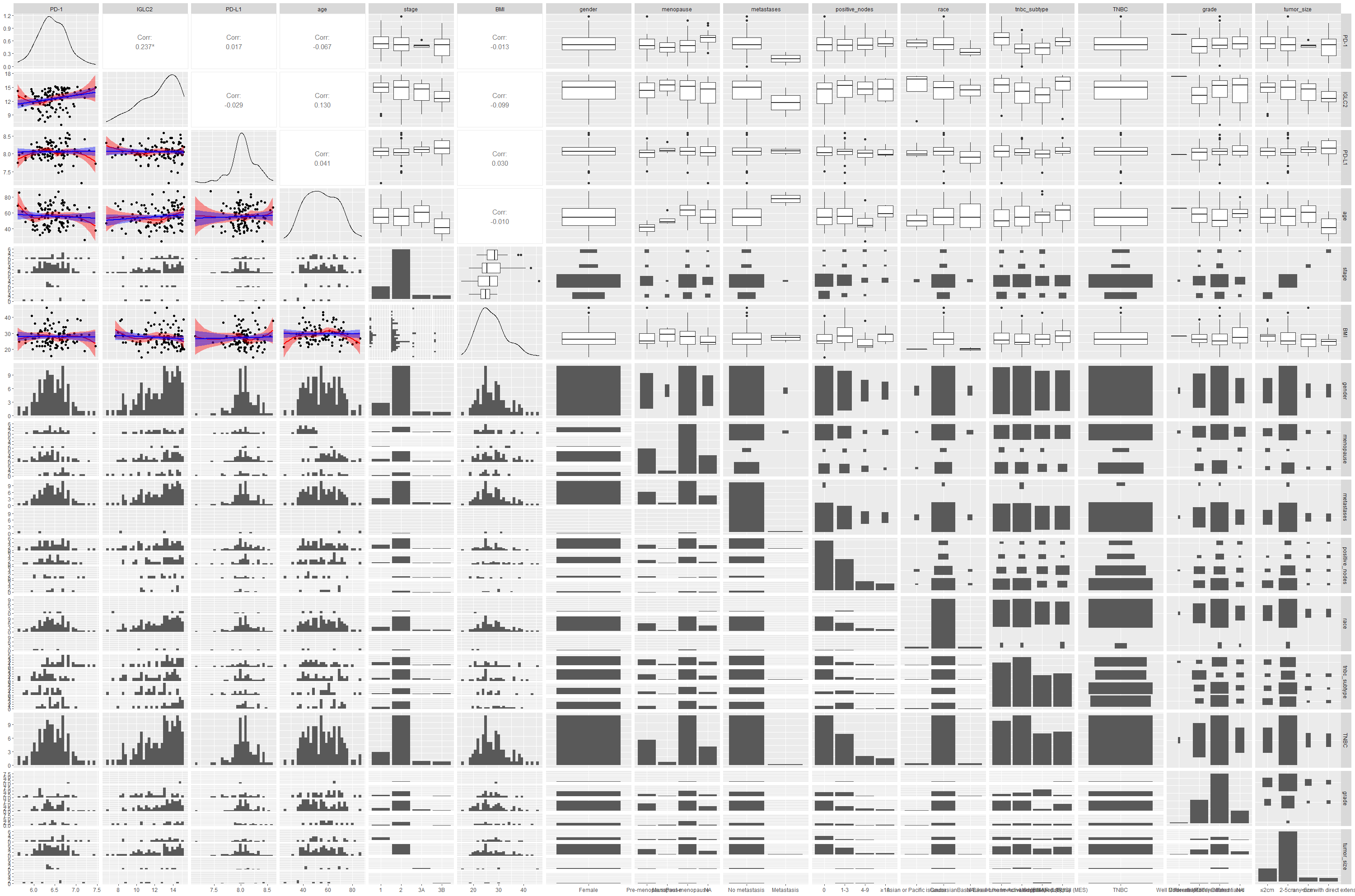


## Figure S 1 Pairwise Scatter plots and boxplots of IGLC2 (marked in red) using GSE76275 [92]. The figure was plotted using R software [93]with the package of ‘GGally’ [92].


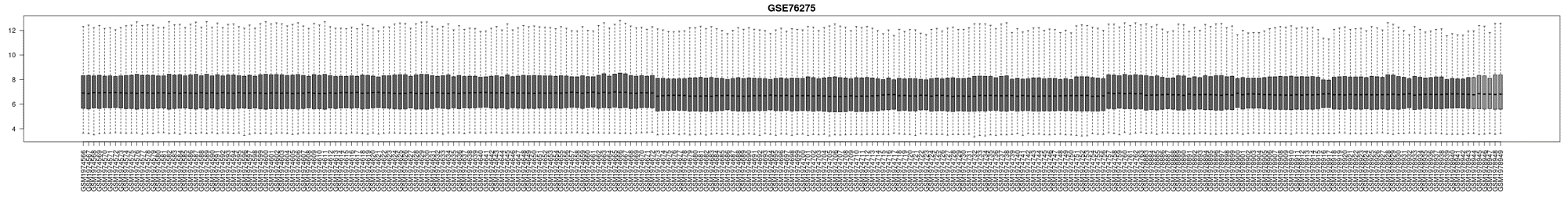


## Figure S 2 The boxplots of log2 transformed gene expression of GSE76275


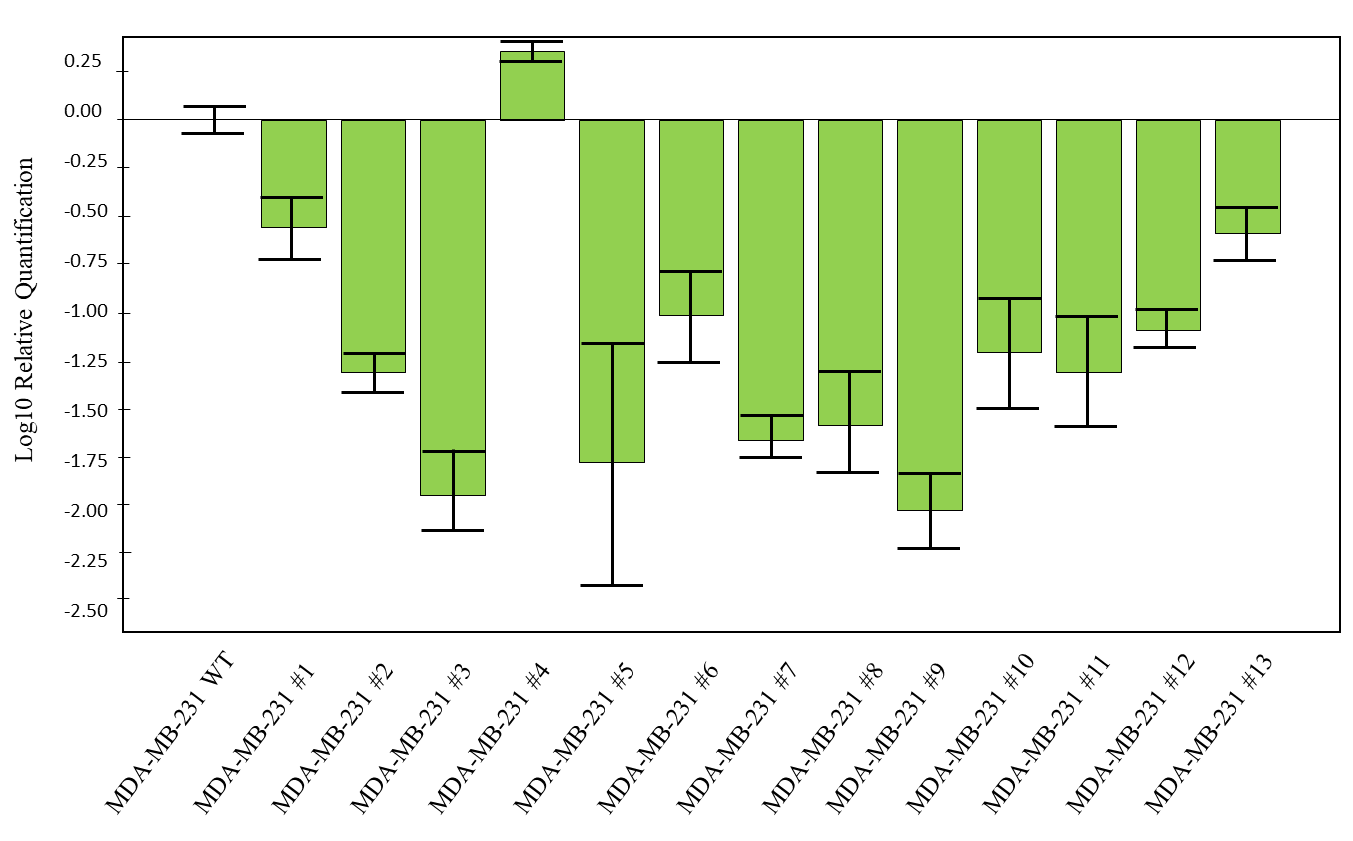


## Figure S 3 The knock-down quantification of IGLC2 in multiple MDA-MB-231 clones. We selected the best one clone to conduct the experiment.

| All TNBC patients | |
| --- | --- |
| 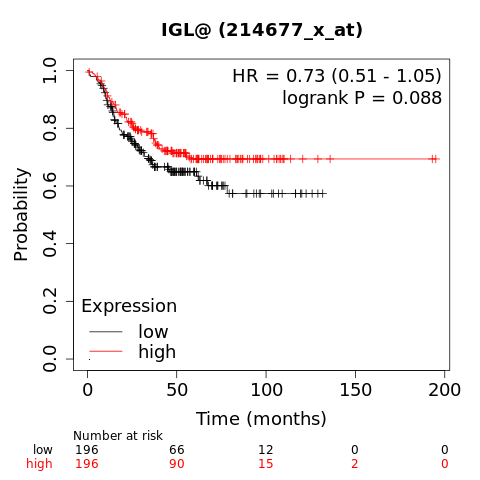 q* = 0.15  n=392  **(A)**RFS | 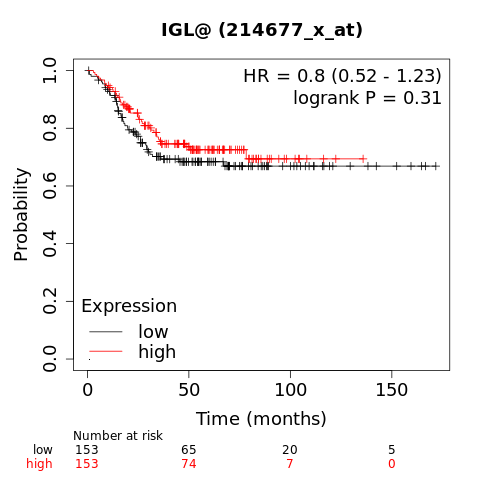 q* = 0.37  n=306  (B)DMFS |
| TNBC patients with Grade 3 | |
| 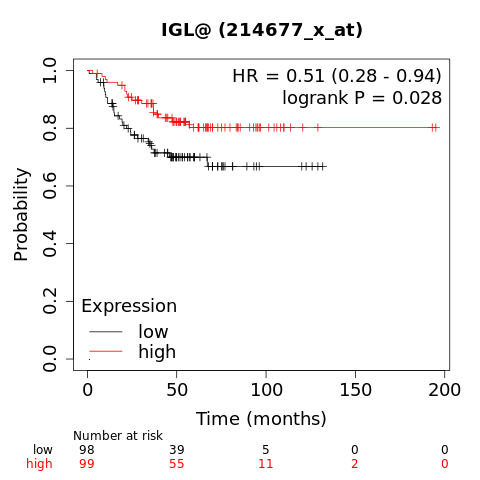 q* = 0.06  n=197  (C) RFS | 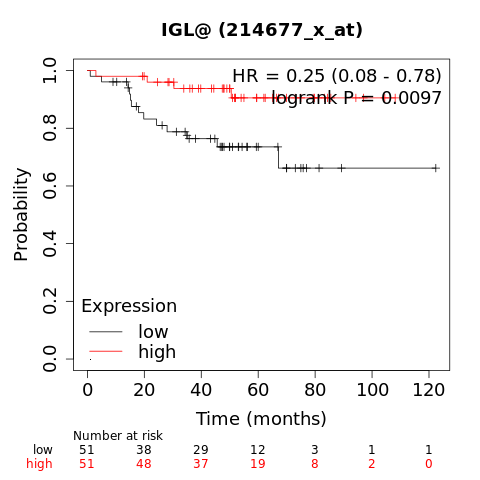 q* = 0.0.3  n=102  (D) DMFS |

| TNBC patients with lymph node negative | |
| --- | --- |
| 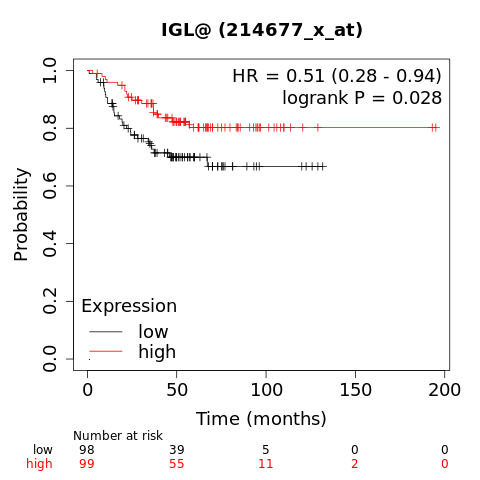 q* = 0.07  n=197  (C) RFS | 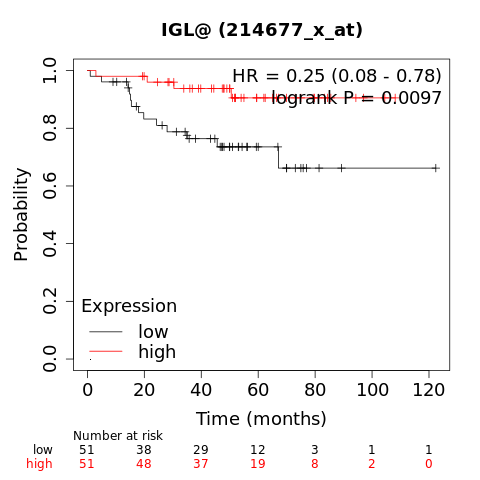 q* = 0.04  n=102  (D) DMFS |

| TNBC patients with lymph node negative and Grade 3 | |
| --- | --- |
| 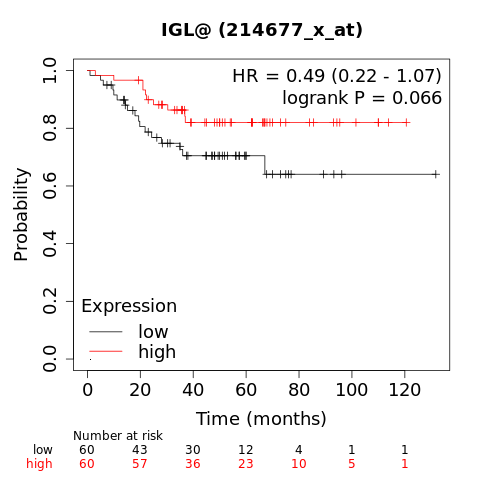 q* = 0.13  n=120  (E) RFS | 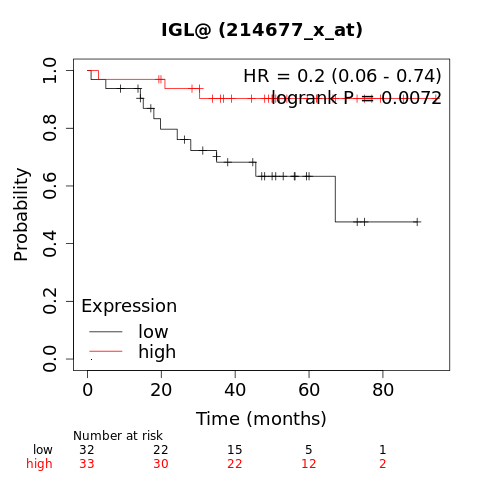 q* = 0.04  n=65  (F) DMFS |

| TNBC patients with lymph nodes positive | |
| --- | --- |
| 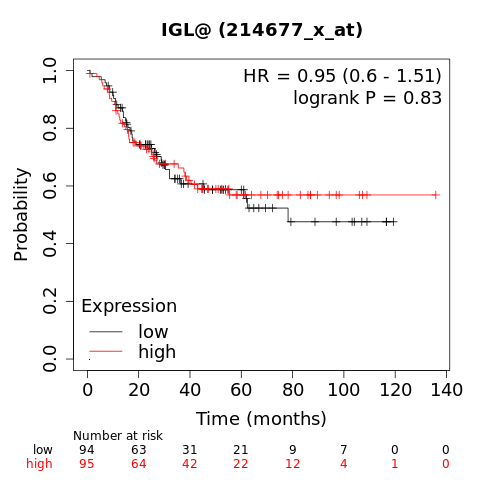 q* = 0.84  n=189  (G) RFS | 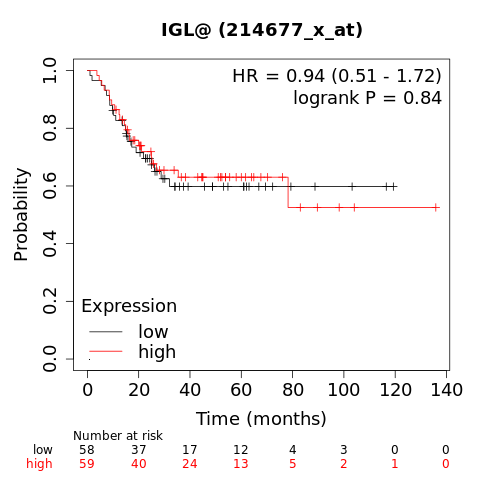 q* = 0.84  n=117  (H) DMFS |
| TNBC patients with Grade 3 | |
| 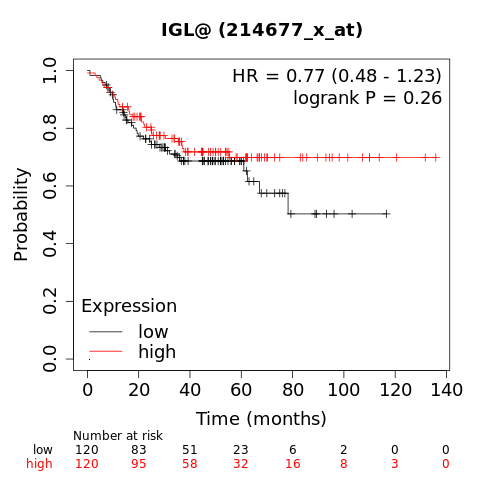 q* = 0.35  n=240  (I) RFS | 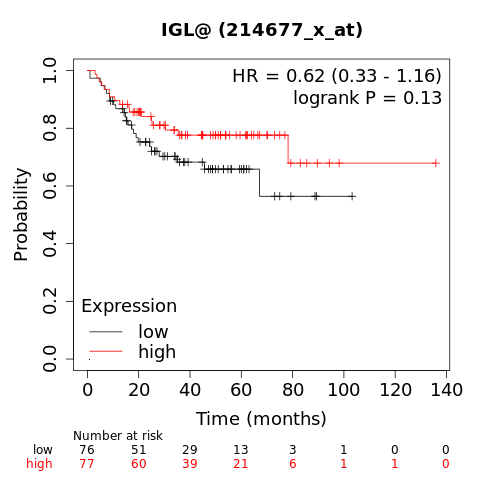 q* = 0.20  n=153  (J) DMFS |

## Figure S 4 Kaplan–Meier analysis of RFS and DMFS of IGLC2 mRNA expression for TNBC subgroups. We used the KM plotter online cancer survival analysis tool (<http://kmplot.com/analysis/>) to evaluate the RFS (A, C, E, G, and I) and DMFS (B, D, F, and H) in TNBC subgroups grouped by grade and lymph node status. The panels (A) and (B) are all from TNBC patients; (C) and (D) are TNBC patients developing negative lymph nodes; (E) and (F) are TNBC patients developing negative lymph nodes and Grade 3; (G) and (H) are TNBC patients developing positive lymph nodes; (I) and (J) are TNBC patients developing Grade 3. The median value was set to be the cut-off point of IGLC2 gene expression. Grade 1 and 2 subgroups were not analyzed given the limited sample size (n < 35) for meaningful analysis. Y axis denotes the probability of RFS or DMFS. * q values were calculated using Benjamini-Hochberg method.


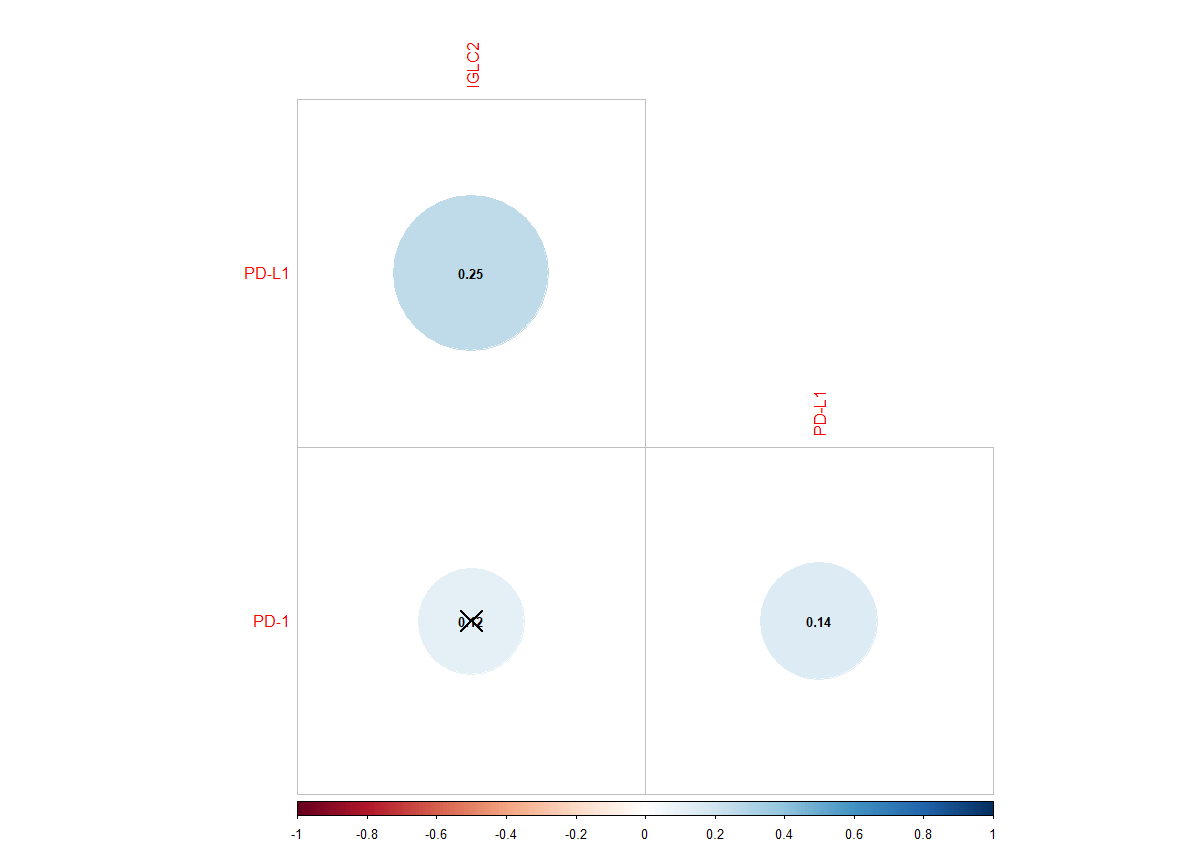


## Figure S 5 Correlation plot of IGLC2, PD-1, and PD-L1 in TNBC tissues from GSE 76275. The cross mark denotes insignificant (p ≥ 0.05) correlation using Pearson correlation analysis. IGLC2 was positively associated with PD-L1 with a correlation coefficient r = 0.25 (p < 0.05).

| All TNBC patients | |
| --- | --- |
| 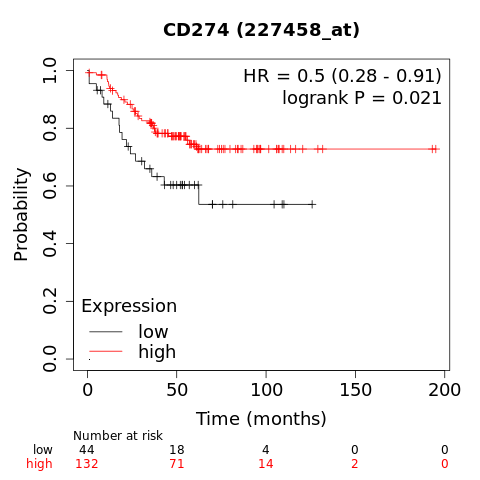 q* = 0.09  n=176  (A) RFS | 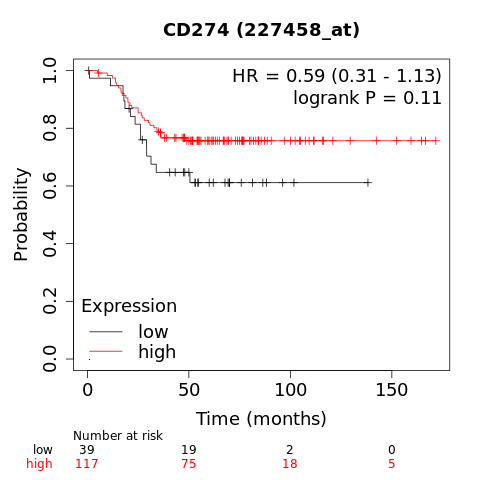 q* = 0.22  n=156  (B) DMFS |
|  | |
|  |  |
| TNBC patients with lymph node negative | |
| 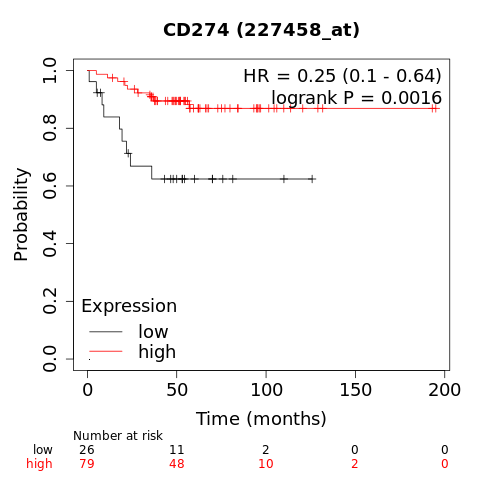 q* = 0.02  n=105  (C) RFS | 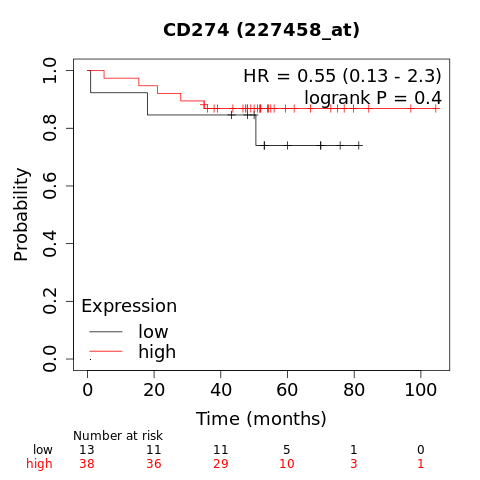 q* = 0.57  n=51  (D) DMFS |

| TNBC patients with lymph node negative and Grade 3 | |
| --- | --- |
| 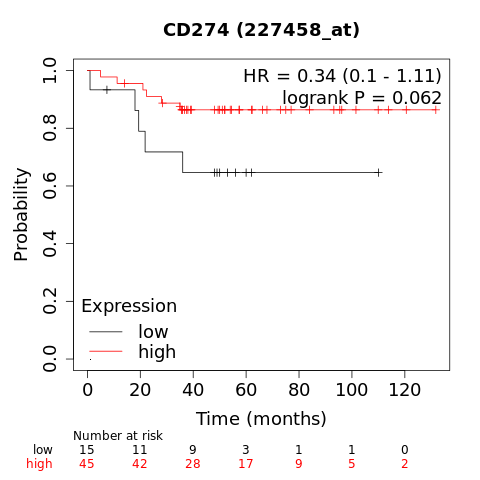 q* = 0.16  n=60  (E) RFS | 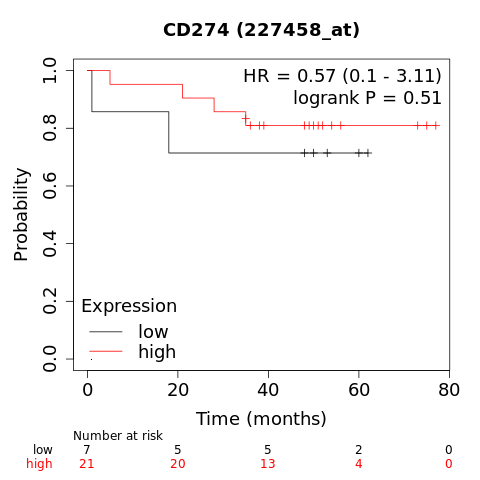 q* = 0.57  n=28  (F) DMFS |

| TNBC patients with lymph nodes positive | |
| --- | --- |
| 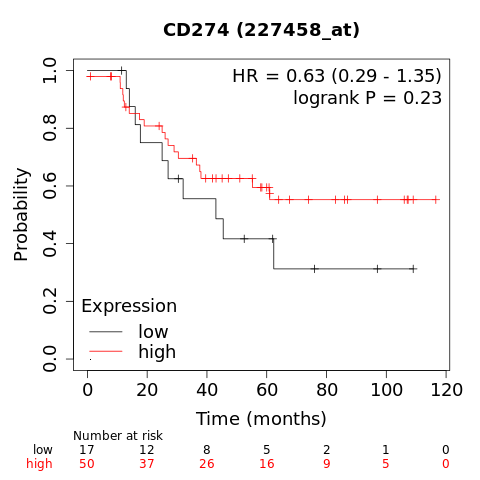 q* = 0.38  n=67  (G) RFS | 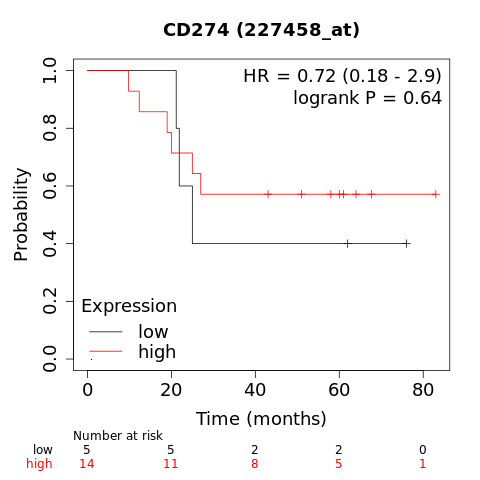 q* = 0.64  n=19  (H) DMFS |

| All TNBC patients with Grade 3 | |
| --- | --- |
| n=92 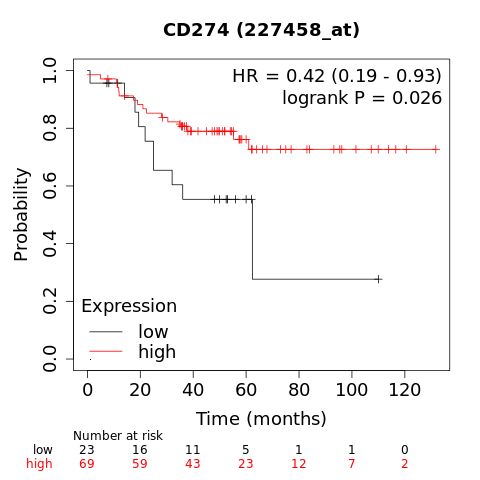 q* = 0.09  (I) RFS | 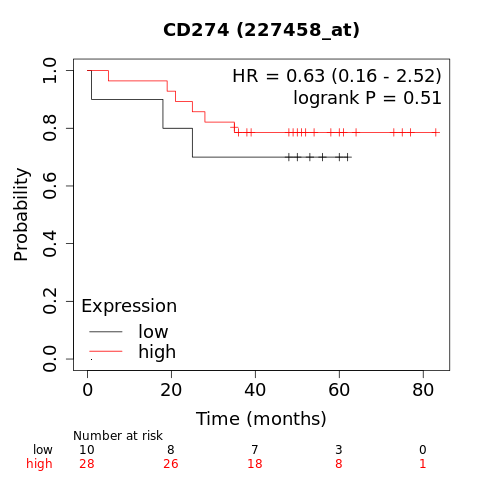 q* = 0.56  n=38  (J) DMFS |

## Figure S 6 Kaplan–Meier analysis of RFS and DMFS of PD-L1 mRNA expression for TNBC subgroups. We used the KM plotter online cancer survival analysis tool (<http://kmplot.com/analysis/>) to evaluate the RFS (A, C, E, G, and I) and DMFS (B, D, F, and H) in TNBC subgroups grouped by grade and lymph node status. The panels (A) and (B) are all from TNBC patients; (C) and (D) are TNBC patients developing negative lymph nodes; (E) and (F) are TNBC patients developing negative lymph nodes and Grade 3 ; (G) and (H) are TNBC patients developing positive lymph nodes; (I) and (J) are TNBC patients developing Grade 3. The lower quartile was set to be the cut-off point of IGLC2 gene expression. Grade 1 and 2 subgroups were not analyzed given the limited sample size (n < 35) for meaningful analysis. Y axis denotes the probability of RFS or DMFS. * q values were calculated using Benjamini-Hochberg method.


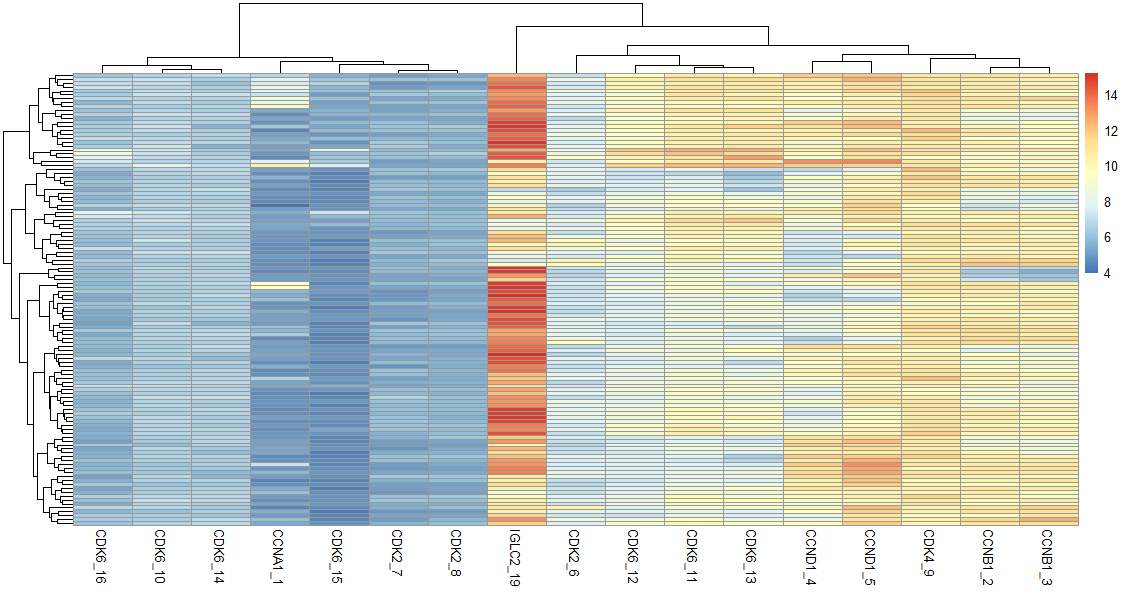


## Figure S 7 The heatmap o IGLC2 and cell cycle related genes (CCNA1, CCNB1, CCND1, CDK2, CDK4 and CDK6) using GSE76275 data set. The mRNA expression was log2 transformed.The figure was plotted using R software [93]with the package of ‘pheatmap’[94].


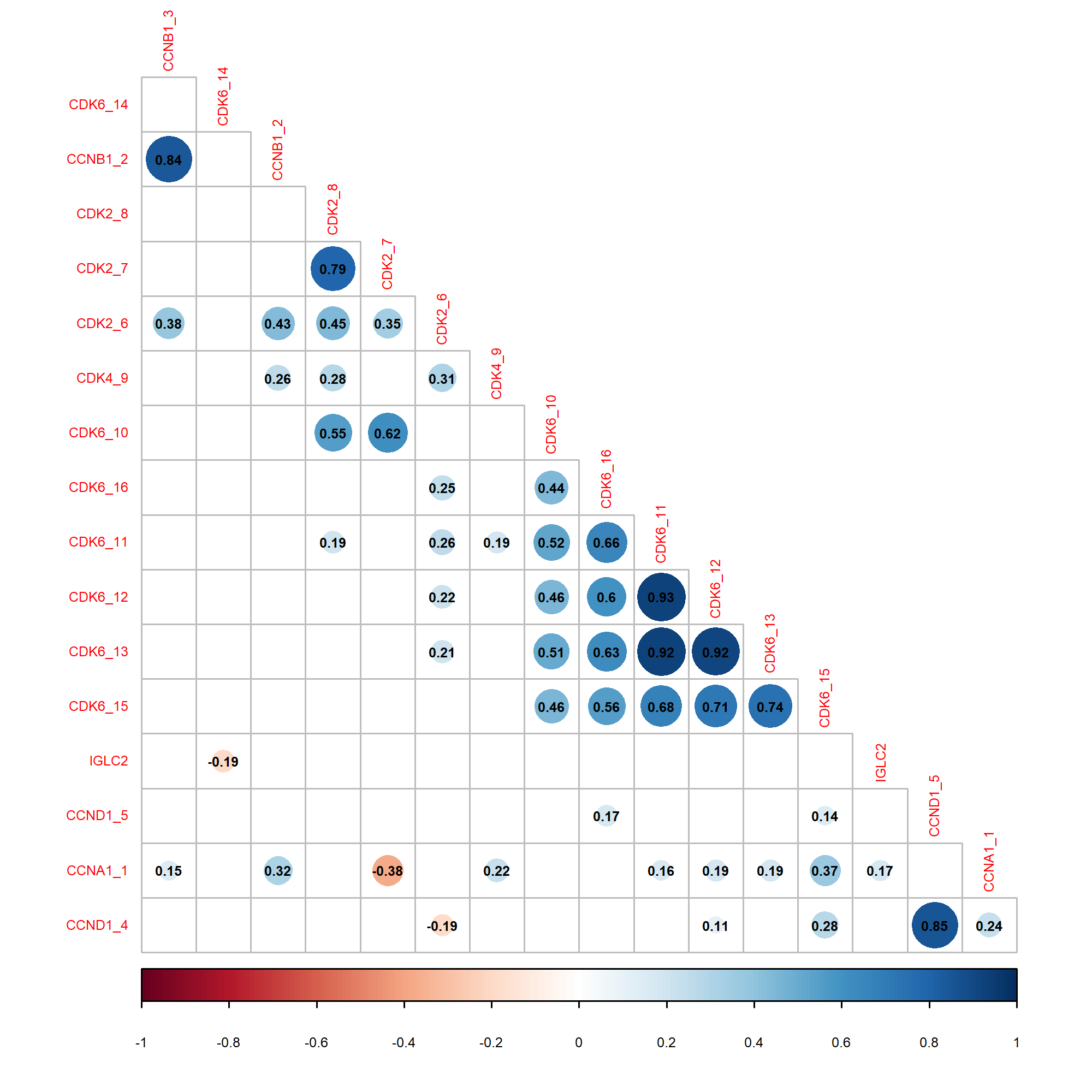


## Figure S 8 The correlation plot of IGLC2 and cell cycle related genes (CCNA1, CCNB1, CCND1, CDK2, CDK4 and CDK6) using GSE76275 data set. The mRNA expression was log2 transformed. Only the significant correlation coefficients (p<0.05) were shown in the figure. The results of IGLC2 was marked with red outlines. The number at the end of each gene symbol served as an identification number for various sequences inside the same gene. The figure was plotted using R software [93]with the package of ‘corrplot’ [95].


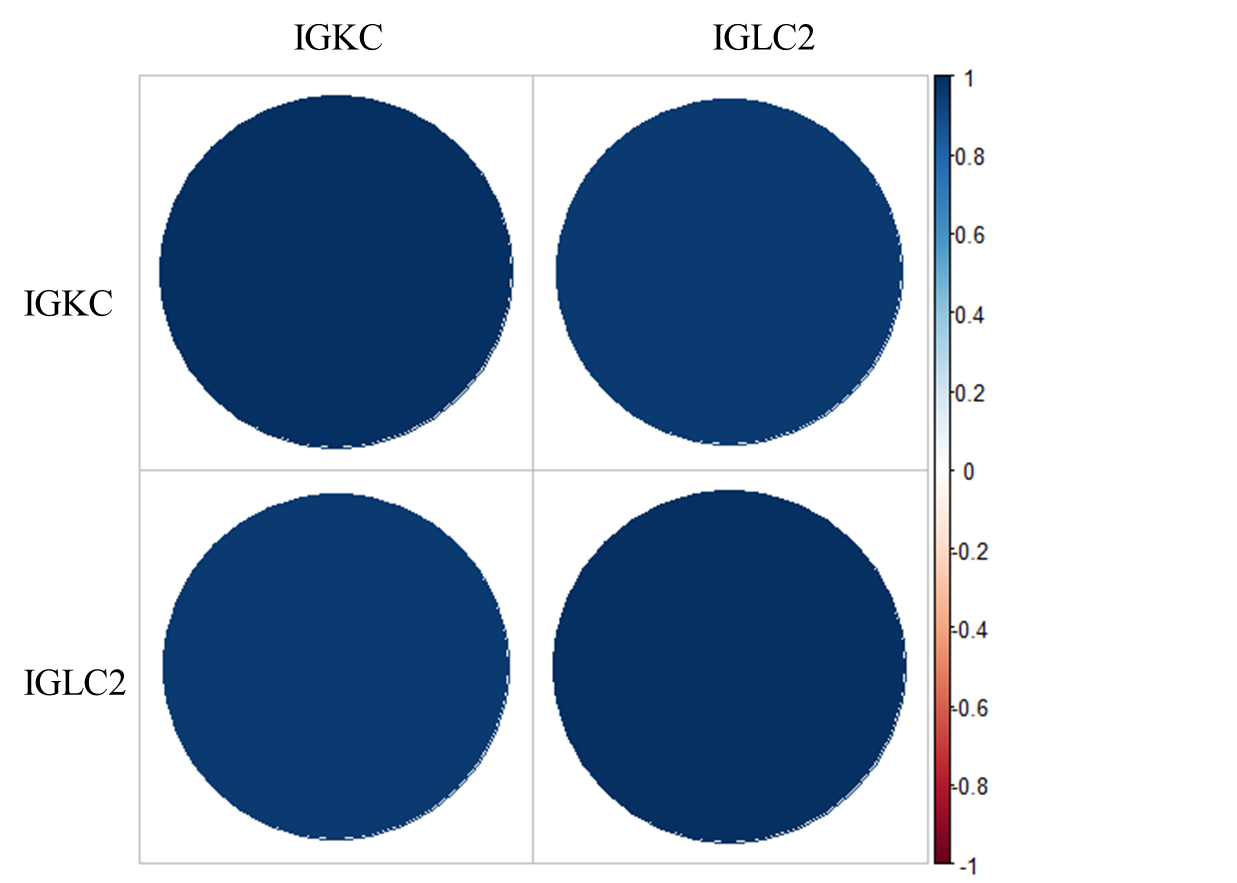


## Figure S 9 Correlation plot of IGLC2 and probe sets of IGKC in TNBC tissues from GSE 76275. The mRNA expressions of IGLC2 and IGKC were highly correlated with each other, denoted by a Pearson correlation analysis with a correlation coefficient r close to 1.


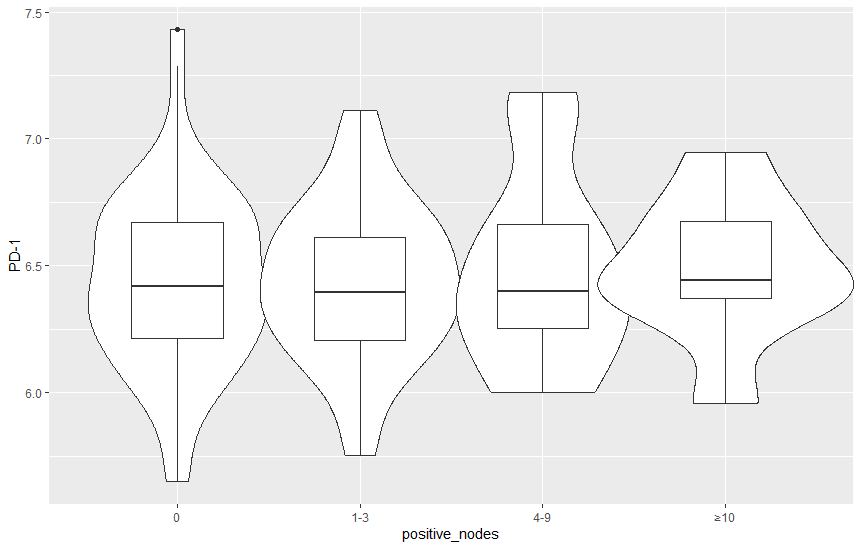


## Figure S 10 Violin boxplots of PD-l log2 mRNA expression in TNBC tissues grouped by positive lymph nodes using the GSE76275 data set
